# GDF11 enhances therapeutic efficacy of mesenchymal stem cells for myocardial infarction via YME1L‐mediated OPA1 processing

**DOI:** 10.1002/sctm.20-0005

**Published:** 2020-06-09

**Authors:** Yun Zhao, Jinyun Zhu, Ning Zhang, Qi Liu, Yingchao Wang, Xinyang Hu, Jinghai Chen, Wei Zhu, Hong Yu

**Affiliations:** ^1^ Department of Cardiology, Second Affiliated Hospital, College of Medicine Zhejiang University Hangzhou Zhejiang Province People's Republic of China; ^2^ Cardiovascular Key Laboratory of Zhejiang Province Hangzhou Zhejiang Province People's Republic of China; ^3^ Pharmaceutical Informatics Institute, College of Pharmaceutical Sciences Zhejiang University Hangzhou Zhejiang Province People's Republic of China; ^4^ Institute of Translational Medicine Zhejiang University Hangzhou Zhejiang Province People's Republic of China

**Keywords:** cardiac, GDF11, mesenchymal stem cells, mitochondria, OPA1

## Abstract

Growth differentiation factor 11 (GDF11) has been shown to promote stem cell activity, but little is known about the effect of GDF11 on viability and therapeutic efficacy of cardiac mesenchymal stem cells (MSCs) for cardiac injury. To understand the roles of GDF11 in MSCs, mouse heart‐derived MSCs were transduced with lentiviral vector carrying genes for both GDF11 and green fluorescent protein (GFP) (MSCs^LV‐GDF11^) or cultured with recombinant GDF11 (MSCs^rGDF11^). Either MSCs^rGDF11^ or MSCs ^LV‐GDF11^ displayed less cell apoptosis and better paracrine function, as well as preserved mitochondrial morphology and function under hypoxic condition as compared with control MSCs. GDF11 enhanced phosphorylation of Smad2/3, which upregulated expression of YME1L, a mitochondria protease that balances OPA1 processing. Inhibitors of TGF‐β receptor (SB431542) or Smad2/3 (SIS3) attenuated the effects of GDF11 on cell viability, mitochondrial function, and expression of YME1L. Transplantation of MSCs^GDF11^ into infarct heart resulted in improved cell survival and retention, leading to more angiogenesis, smaller scar size, and better cardiac function in comparison with control MSCs. GDF11 enhanced viability and therapeutic efficiency of MSCs by promoting mitochondrial fusion through TGF‐β receptor/Smad2/3/YME1L‐OPA1 signaling pathway. This novel role of GDF11 may be used for a new approach of stem cell therapy for myocardial infarction.

## Graphical abstract

Growth differentiation factor 11 (GDF11) induces fusion of mitochondrial networks in hypoxic mesenchymal stem cells (MSCs) through ALK4/5‐p‐Smad2/3 pathway. Increased YME1L rebalances OPA1 processing and inhibits mitochondrial fragmentation in the MSCs under hypoxia stress condition. This study suggests that YME1L promotion induced by GDF11 can serve as a potential approach to protect MSCs under hypoxia condition, and improve their therapeutic effect for myocardial infarction

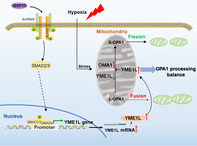

## 1 INTRODUCTION

Growth differentiation factor 11 (GDF11) is a member of the transforming growth factor‐β (TGF‐β) superfamily that plays an essential role in mammalian development. GDF11 can affect the developmental process via exerting its effects at least partially by binding to TGF‐β activin receptors type I (TGFβR1/ALK5) and type II (ACVR2A, ACVR2B).^1^ GDF11 has been shown to stimulate proliferation and angiogenesis of stem cells in ischemia/reperfusion models and to be implicated in the regulation of mature cell and adult organ in a variety of pathological conditions.^2^, ^3^ However, little is known about the physiological functions of GDF11 in mesenchymal stromal cells (MSCs), which possess mesenchymal stromal characteristics with classic mesenchymal stromal markers. Recent research showed that MSCs play a critical role in maintaining normal cardiac function, as well as in cardiac remodeling during pathological conditions.^4^, ^5^, ^6^ However, therapeutic effect on myocardial infarction by MSCs is limited due to the poor survival rate given a hostile microenvironment in an ischemic heart. This study aims to investigate a novel strategy whether GDF11 could offer heart‐derived cardiac MSCs higher viability and greater therapeutic potential for the repair of an ischemic heart.

Mitochondria serve as a key regulator of cellular metabolic activity, and play a critical role in cell death and development of diseases.^7^ Accumulating evidence revealed that mitochondrial dynamic homeostasis is important for cell survival and activity, which is mainly operated by mitochondrial dynamics‐related proteins.^8^, ^9^, ^10^ Unbalanced mitochondrial fusion and fission can lead to mitochondrial fragmentation, which occurs in the status of stress and/or diseases. Studies showed that optic atrophy 1 (OPA1) is responsible for the fusion and fission in the inner mitochondrial membrane. OPA1 can undergo cleavage and form different isoforms, ranging from long form (L‐OPA1) to short isoform (S‐OPA1).^11^ The different isoforms of OPA1 serve as mitochondrial energetic maintenance proteins, with L‐OPA1 mainly responsible for mitochondrial fusion, while the S‐OPA1 being involved in the acceleration of mitochondrial fission.^12^ Thus, loss of such OPA1 isoforms balance can interrupt mitochondrial dynamics, perturb cristae structure, and hence increase the susceptibility of cells toward apoptosis. It is being recognized that a well‐organized cleavage of OPA1 by YME1L and OMA1 at two distinct sites leads to a balanced accumulation of both L‐OPA1 and S‐OPA1 and maintains normal mitochondrial morphology.^13^, ^14^ The role of GDF11 on mitochondrial dynamic homeostasis has yet been studied.

In the present study, we hypothesized that GDF11 plays an important role in mitochondrial homeostasis of cardiac MSCs, which accounts for the enhanced therapeutic effects of MSCs. Our results demonstrated that GDF11 conferred mitochondrial integrity of MSCs mainly by activating activin receptor‐like kinase (ALK) 5 and Smad2/3 signaling pathway to balance the ratio of L‐OPA1 over S‐OPA1, resulting in much improved cardiac function.

## 2 MATERIALS AND METHODS

### 2.1 Cell isolation and culture

All animal experiments were performed with the approval of the Animal Ethics Committee of Zhejiang University. Mouse cardiac MSCs were isolated from heart tissues of mouse (C57BL/6) at 8 to 12 weeks by procedure described in previous studies,^15^, ^16^ and were provided by Dr Yaoliang Tang at Medical College of Georgia, Augusta University (Augusta, Georgia). Briefly, ventricular heart tissues were minced and digested with collagenase IV and dispase, and then cultured. The grown out cells migrated from adherent explants were collected and undergone hematopoietic cell depletion by magnetic activated cell sorting. These cells were cultured normally under 21% O_2_ and 5% CO_2_ at 37°C in complete medium: Dulbecco's modified Eagle medium (DMEM)/F12 containing 10% fetal bovine serum (FBS), 200 mmol/L l‐glutamine, 55 nmol/L β‐mercaptoethanol and 1% MEM nonessential amino acid, and used as MSCs for the whole study. MSCs were characterized by flow cytometry analysis for surface markers CD44, CD105, CD29, CD45, FLK‐1, and CD31 (Figure S1).

For hypoxia culture, MSCs were plated in a six‐well plate at a density of 1 × 10^6^ cells per well with complete growth medium. The culture media were replaced with 2 mL of serum‐free growth media before hypoxia treatment. Hypoxia (0.5% O_2_, 5% CO_2,_ 37°C) treatment was achieved with a ProOx‐C‐chamber system (Biospherix, Redfield, New York) for 48 hours. Cell proliferation was examined using 5‐ethynyl‐2′‐deoxyuridine (EdU) staining. Cell viability was examined using Cell Counting Kit‐8 (CCK8) assay.

MSCs were transduced with lentiviral vector carrying full‐length mouse GDF11 tagged with flag (Flag‐GDF11) and green fluorescent protein (GFP) to generate MSCs^LV‐GDF11^ overexpressing GDF11 (Figure S2A). MSCs that were transduced with lentiviral vector without GDF11 gene (MSCs^LV^) were used as control. MSCs were also cultured with recombinant GDF11 (rGDF11, 50 ng/mL) (Peprotech, Rocky Hill, Connecticut) for 24 hours (MSC^rGDF11^) for the specified analysis. Silence interference RNA (siRNA) targeting mouse GDF11 (si‐GDF11), OPA1 (si‐OPA1), and YME1L (si‐YME1L) and control scrambled siRNA (si‐NC) were synthesized by Guangzhou RiboBio Co., Ltd. (Guangzhou, China). siRNA transfection was conducted using Lipofectamine 3000 (Invitrogen). The effects of downregulation of the target genes (GDF11, OPA1, YME1L) are shown in Figure S2B‐D. The gene primers are shown in Table S1.

### 2.2 Preparation of MSCs‐conditioned medium and measurement of vascular endothelial growth factor A

Conditioned medium was generated as previously described.^17^ Specified MSCs were grown in DMEM with 10% FBS. As specified, either rGDF11 (50 ng/mL), or TGF‐β type I receptor inhibitor SB431542 (1 nm) or Smad3 inhibitor SIS3 (3 nm) was added into the medium 24 hours prior to medium change. The medium was replaced with fresh DMEM/F12 without serum. After cultured for another 48 hours, the supernatants were collected. Medium was then concentrated 5‐fold by Centricon concentrators. Vascular endothelial growth factor (VEGF) in the concentrated medium was determined using a commercial ELISA kit. Matrigel assay was used to analyze the ability of MSCs‐conditioned media to promote tube formation of HUVECs in vitro. The conditioned medium had been normalized by an equivalent number of MSCs (1 × 10^6^ cells).

### 2.3 Transmission electron microscopy

Transmission electron microscopy (TEM) was applied to detect the mitochondrial network ultrastructure of MSCs and was performed as described previously^18^ using a Hitachi Model H7650 transmission electron microscope. The images were obtained randomly to measure the mitochondrial ultramicrostructure using ImageJ (NIH) under magnification of ×10 000 and ×26 500.

### 2.4 Oxygen consumption rate

Oxygen consumption rate (OCR) was measured in the intact cells by using OROBOROS Oxygraph‐2k at 30°C as described previously.^18^ The mitochondrial inhibitors used were ATP synthase inhibitor oligomycin (final concentration: 1 μg/mL), proton ionophore carbonylcyanide‐p‐trifluoromethoxyphenylhydrazone (FCCP; final concentration 1 μM), and antimycin A, a complex III inhibitor, and rotenone, a complex I inhibitor (final concentration, 1 μM). Mitochondrial function parameters, including basal respiration, ATP turnover rate, proton leak, and maximal and spare respiratory capacity, were determined by using mitochondrial inhibitors.

### 2.5 ATP measurement

Total cellular ATP content of MSCs under hypoxic condition was determined by using a luminescence ATP detection kit in accordance with the manufacturer's instructions (Beyotime Biotechnology, Shanghai, China).

### 2.6 Mitochondrial membrane potential

Cells were stained with tetramethyl rhodamine methyl ester (TMRM, 200 nmol/L) for 30 minutes at 37°C in a humidified incubator with 5% CO_2_, in the presence or absence of FCCP (50 μM) or oligomycin (10 μM), which serves as positive and negative controls, respectively. Images were acquired under a fluorescence microscope (×400) at 549 nm for excitation and 573 nm for emission. Mitochondrial membrane potential was measured by quantifying TMRM fluorescent intensity.

### 2.7 Isolation of cytosolic and mitochondrial fractions

Isolation of cytosolic and mitochondrial fraction was conducted by using a Mitochondria Isolation Kit for Cultured Cells (Beyotime Biotechnology, Shanghai, China) following the manufacturer's protocol. In brief, 3 × 10^6^ cells with indicated treatments were collected by centrifuging cell suspension, and then mitochondria isolation reagent with phenylmethylsulfonyl fluoride was added into the cell pellets. The cell resuspension was homogenized by a glass homogenizer, centrifuged at 600*g* for 10 minutes at 4°C. The supernatant was transferred to a new tube and centrifuged at 11 000*g* for 10 minutes at 4°C. The supernatant was transferred to a new tube, and the pellet contained the isolated mitochondria. Finally, cytosolic fraction was derived from the supernatant centrifuged at 12 000*g* for 10 minutes at 4°C. Purity of mitochondria was ensured by detecting the expression of tubulin that was the loading control of cytosolic protein.

### 2.8 Chromatin immunoprecipitation assay

Promoter of YME1L was analyzed using the Jaspar software (http://jaspar.genereg.net/) to identify putative binding sequences by Smad2/3. Chromatin immunoprecipitation (ChIP) was performed using Simple ChIP Plus Sonication Chromatin IP Kit (Cell Signaling Technology) according to the manufacturer's instruction with Smad2/3 antibody or rabbit IgG as an isotype control. Approximately 1 × 10^7^ cells were evaluated for each sample. The purified DNA and input genomic DNA were analyzed by real‐time PCR. The PCR products were analyzed by gel electrophoresis. Primer sequences of YME1L promoter are shown in Table S1.

### 2.9 Murine myocardial infarction and cell delivery

Myocardial infarction (MI) model was performed on male mice (C57BL/6, 8‐10 weeks old, 20‐25 g weight). MI surgery was conducted by ligation of left anterior descending coronary artery as described previously.^19^ MSCs^LV^ or MSCs^LV‐GDF11^ (both 5 × 10^5^ cells, in 20 μL DMEM) or DMEM alone were injected at five sites around the border zone of infarct heart immediately after the ligation using a 31‐gauge Hamilton syringe. Sham group underwent the same surgical procedures except for the permanent ligation step. The experiments were randomized into double‐blind groups.

### 2.10 Histochemical staining

Heart tissues were harvested at day 3 and 28 post‐MI for histological analysis as described previously.^17^ Scar size was evaluated by Sirius Red staining of heart tissues recovered 28 days post MI. The retained MSCs at day 3 post‐MI and capillaries and small arteries at day 28 post‐MI were identified with immunofluorescence staining using specific antibodies (GFP, CD31, and α‐SMA, respectively). Positively stained cells were counted in three sections per heart, five high‐power fields (HPFs) per section under microscopy.

### 2.11 Statistical analysis

The experiments were randomized into double‐blind groups. All in vitro experiments were conducted with at least three independent biological replications. All data are presented as means ± SD. Statistical analyses were performed with Prism 6 (GraphPad Software Inc). Continuous variables were compared by Student's *t*‐test. All additional normally distributed data were analyzed by one‐way ANOVA vs control group followed by Holm‐Sidak post hoc analysis. Presented data meet the assumptions of the tests. The value of *P* <.05 was considered significant.

Detailed information was listed in Online Resource.

## 3 RESULTS

### 3.1 GDF11 protects cardiac MSCs from apoptosis under hypoxic condition in vitro

**FIGURE 1:**
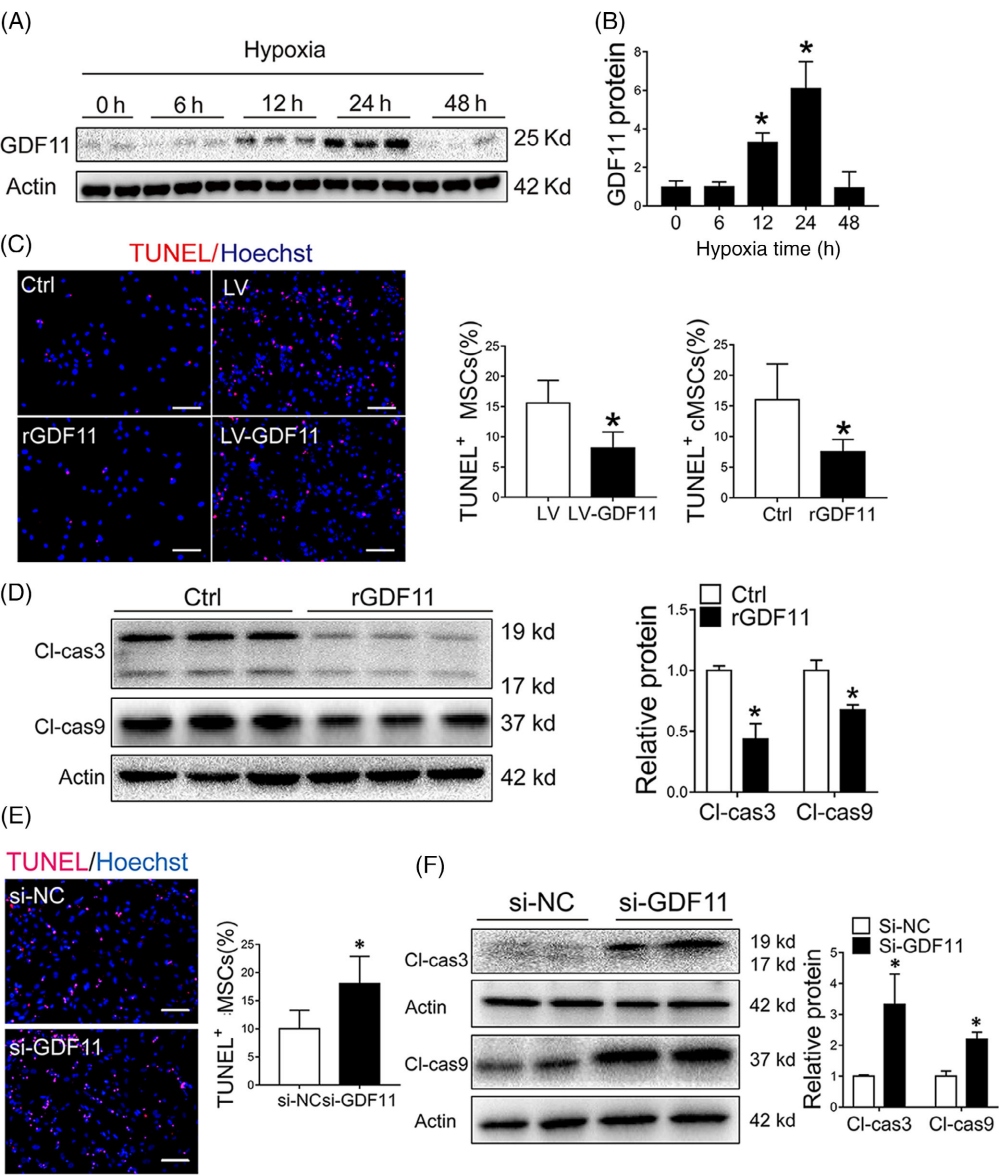
GDF11 protected MSCs against apoptosis under hypoxia condition in vitro. A, GDF11 expression in MSCs were detected by Western blot under normoxia and hypoxia condition at specified times, and β‐actin served as a loading control. B, Quantification of relative GDF11 protein level (n = 3). C, MSCs were pretreated with rGDF11 or overexpressed GDF11 by viral transduction (LV‐GDF11). Representative TUNEL staining images of Control (Ctrl), rGDF11, LV (vector control) and LV‐GDF11 were captured. Scale bar = 50 μm. Quantification of apoptotic cells was presented as ratio of TUNEL‐positive nuclei over the total nuclei from 8 to 10 randomly selected fields in each sample. D, Cleaved caspase 3 (Cl‐cas3) and cleaved caspase 9 (Cl‐cas9) proteins in MSCs pretreated with rGDF11 were assessed by Western blot and quantified by densitometry (n = 3). E, Representative TUNEL staining images of MSCs transfected with siRNA GDF11 (si‐GDF11) or siRNA control (si‐NC). Scale bar = 50 μm. Quantification of apoptotic cells was presented as ratio of TUNEL‐positive nuclei over the total nuclei from 8 to 10 randomly selected fields in each sample. F, Cleaved caspase 3 and 9 proteins of MSCs after transfected with si‐NC and si‐GDF11 were assessed by Western blot; and protein expression levels were quantified by densitometry (n = 4). Data are shown as mean ± SD. *P < .05 vs Ctrl/si‐NC

To examine the protective effect of GDF11 on MSCs, expression of GDF11 in MSCs under hypoxic condition was measured. Expression of GDF11 at protein level was significantly increased during initial 24 hours in hypoxic culture condition, which then decreased at 48 hours (Figure 1A,B), indicating a potential role for GDF11 in hypoxic condition.

Then, the effect of GDF11 on MSC proliferation and viability was examined by adding external GDF11 (MSCs^rGDF11^) or transduction of MSCs with lentiviral vector carrying GDF11 gene (MSCs^LV‐GDF11^). There was no significant difference of MSC proliferation under normoxic condition when rGDF11 was added to the culture medium (Figure S3A). On the other hand, less apoptotic cells were observed in MSCs^rGDF11^ or MSCs^LV‐GDF11^ as compared with control MSCs after they were cultured under hypoxic condition (Figure 1C), which was consistent with expression levels of cleaved caspase 3/9 (Figure 1D). Cell viability was also higher in MSCs^rGDF11^ than the in the controls (Figure S3B). The protective effect of GDF11 was diminished when MSCs were transfected with siRNA of GDF11 (MSCs^si‐GDF11^) (Figure 1E,F), for which a decreased GDF11 activity was detected (Figure S2). Taken together, the data showed that GDF11 had protective effect on MSCs under hypoxic condition.

### 3.2 GDF11 enhanced paracrine effects of MSCs in vitro

**FIGURE 2:**
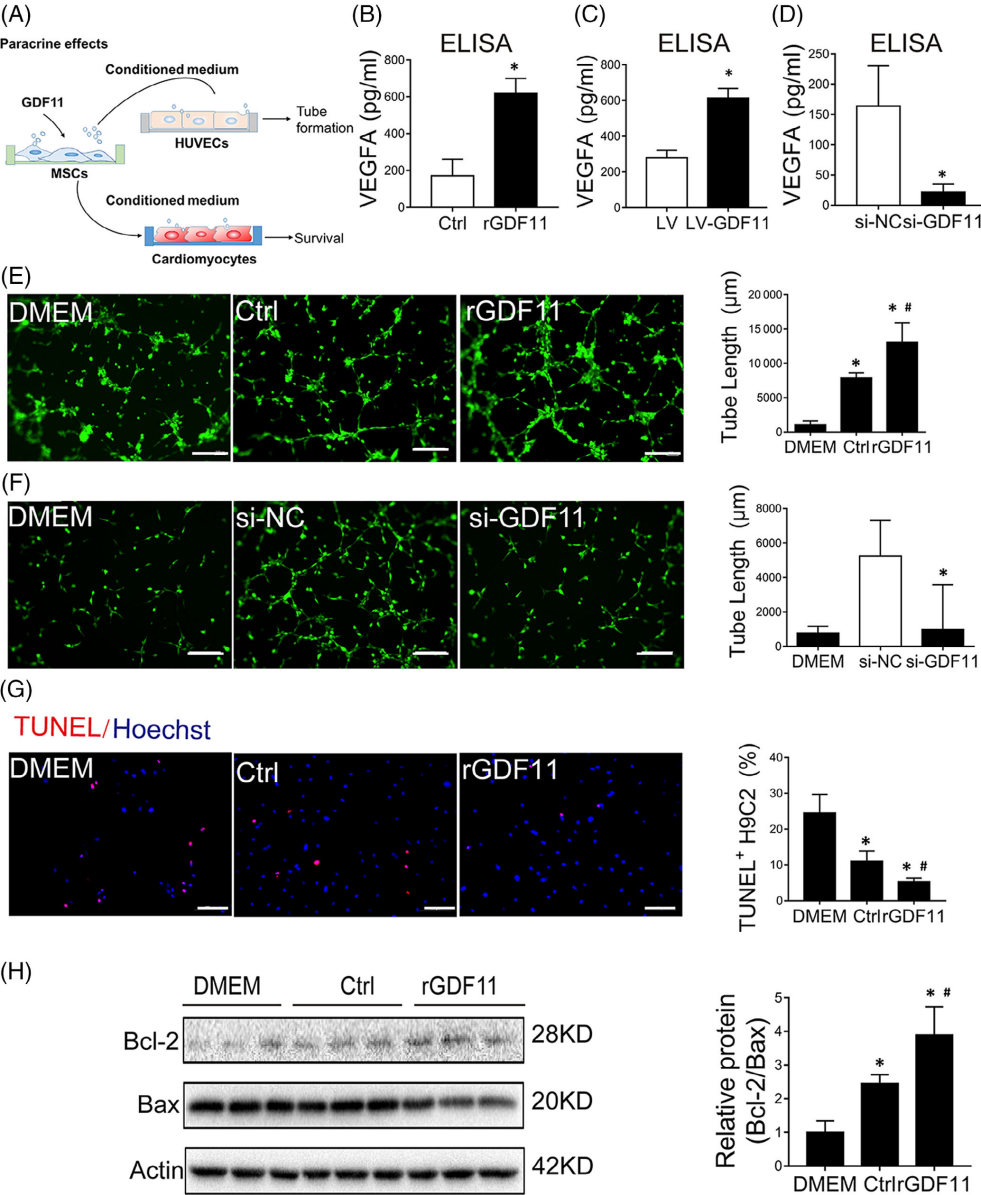
GDF11 enhanced paracrine effects of MSCs in vitro. A, Schematic working showed the experiments for evaluating the paracrine effect of MSCs with different treatment. B‐D, The VEGFA levels in conditioned medium from MSCs (Ctrl, rGDF11) (B) or (LV, LV‐GDF11) (C) and (si‐NC, si‐GDF11) (D) were determined by ELISA assay. E, Representative images of tube formation assay of HUVECs/GFP under fluorescent microscopy. HUVECs were cultured with DMEM or conditioned medium of MSCs that had been cultured in the absence (Control) or presence of rGDF11. Scale bar = 100 μm. Quantification of tube formation was shown in bar graphs (n = 8). F, Representative images of tube formation assay of HUVECs/GFP that were cultured with DMEM or conditioned medium of MSCs that had been transfected with siRNAs (si‐GDF11, and NC as negative control). Quantification of tube formation was shown in bar graphs (n = 13). Scale bar = 100 μm. G, TUNEL assay of apoptosis of H9C2 cells that were cultured with DMEM medium or conditioned medium of MSCs that had been cultured in the absence (Control) or presence of rGDF11 (n = 8). Scale bar =50 μm. H, Western blot analysis of apoptosis‐related proteins in H9C2 cells cultured as in G. β‐actin was used as internal control. The quantification was shown in bar graphs (n = 3). The conditioned medium had been normalized by an equivalent number of MSCs (1 × 10^6^ cells). Data are shown as mean ± SD. *P < .05 vs DMEM/Ctrl/si‐NC, ^#^ *P <* .05 vs Ctrl

Given that GDF11 can improve MSC survival under hypoxic condition, we then tested whether GDF11 can enhance paracrine effects of MSCs. Endothelial cells (ECs) or cardiomyocytes were cultured with the conditioned media from different MSCs (Figure 2A). VEGFA levels in the conditioned media from either MSC^rGDF11^ (Figure 2B) or MSC^LV‐GDF11^ (Figure 2C) were significantly higher as compared with that from control MSCs, while VEGF was downregulated when MSCs were treated with specific siRNA for GDF11 (Figure 2D). Accordingly, tube formation of human umbilical vein ECs (HUVECs) was significantly augmented by the media from either MSC^rGDF11^ (Figure 2E) or MSC^LV‐GDF11^ (Figure S4) as compared with medium from control MSCs. As noted, the conditioned media from control MSCs had a better effect on promoting tube formation of HUVECs than just DMEM medium, confirming the paracrine effect of MSCs.

Such effect of protube formation of HUVECs by the condition media from MSCs was diminished when MSCs were transfected with si‐GDF11 (Figure 2F). When conditioned medium of cMSC^rGDF11^ was used to culture cardiomyocyte H9C2 under hypoxia for 48 hours, the number of apoptotic H9C2 cells were significantly less than the cells cultured with DMEM or conditioned medium from control MSCs (Figure 2G). Being consistent with this, the ratio of Bcl2/Bax as an antiapoptosis indication was significantly increased (Figure 2H). These results indicate that GDF11 can augment paracrine effects of MSCs.

### 3.3 GDF11 protected morphology and function of mitochondria in MSCs under hypoxic condition

**FIGURE 3:**
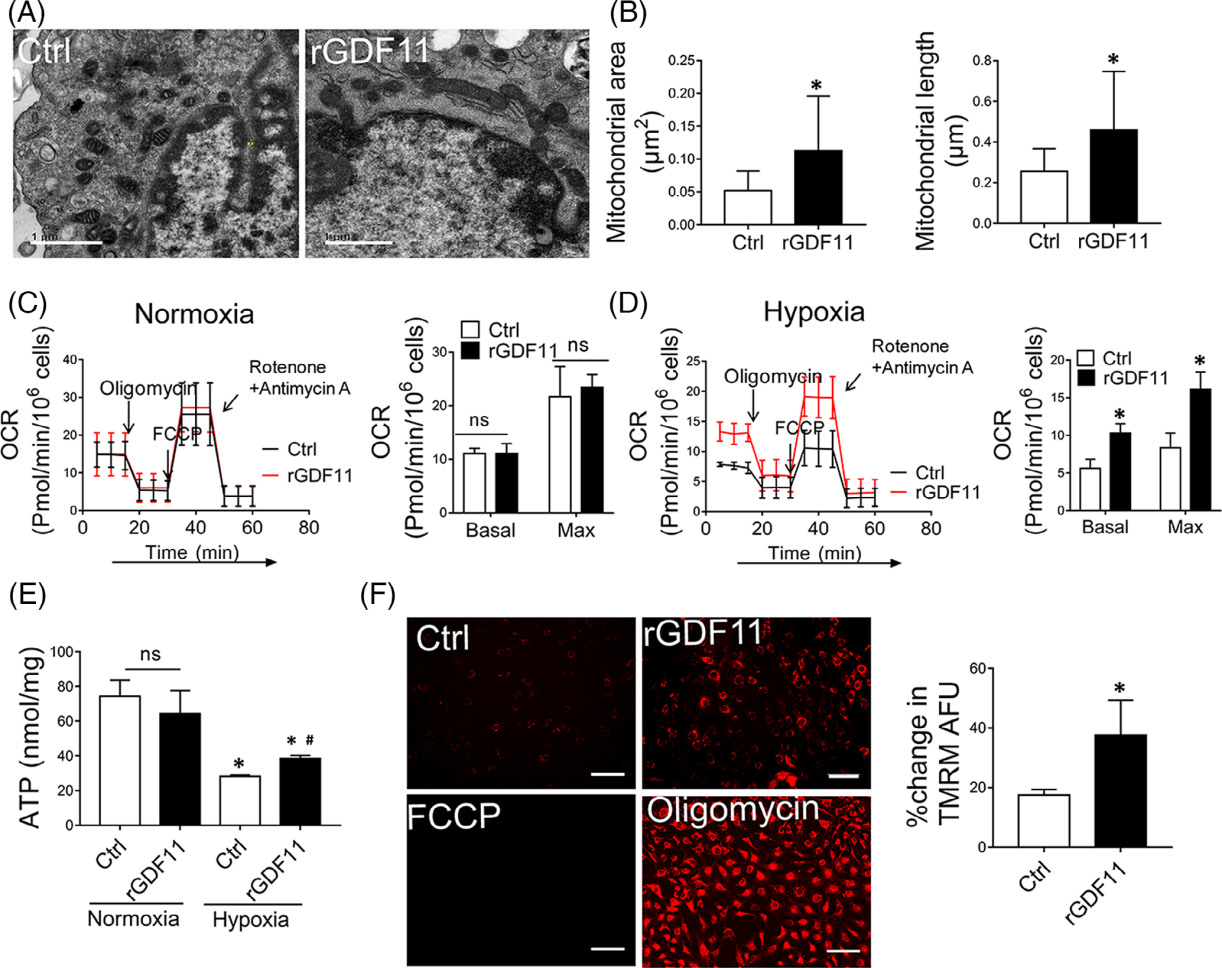
GDF11 protected mitochondrial morphology and function under hypoxic condition. A, Representative images of MSCs under hypoxia condition were taken by electron microscopy (×10 000). Scale bar =1 μm. B, Mitochondrial morphology was analyzed by quantification of area and longitudinal length of mitochondria in MSCs^Ctrl^ and MSCs^rGDF11^ (n = 77 for MSCs^Ctrl^, and n = 95 for MSCs^rGDF11^). C, D, Mitochondrial respiration reflected by OCR was detected in MSCs alone, MSCs treated with rGDF11 under normoxic (C) or hypoxic (D) conditions. OCRs were assayed under both basal and maximal conditions (n = 3). E. Cellular ATP levels of MSCs^Ctrl^ and MSCs^rGDF11^ under normoxic or hypoxic conditions were measured through luciferin/luciferase‐based assay, and the data were calibrated with protein content. F, Images of MSCs stained with TMRM under fluorescence microscope were used to measure mitochondrial membrane potential. MSCs were treated with rGDF11 (50 ng/mL) for 24 hours then exposed to hypoxia conditions for 48 hours. MSCs treated with either FCCP (50 μmol/L) or oligomycin (10 μmol/L) were served as negative and positive controls, respectively. Scale bar = 100 μm. Bar graph shows relative mean of fluorescence intensity (MFI) of Control or rGDF11 divided by the difference of MFI between Oligomycin and FCCP (n = 8). Data were shown as mean ± SD. *P < .05 vs Ctrl, ^#^ *P <* .05 vs Hypoxia Ctrl

To investigate the underlying mechanism through which GDF11 improved the activities of MSCs, we first examined mitochondrial morphology by using TEM. Under normoxic condition, rGDF11 had no significant effects on mitochondria of MSCs (Figure S5). After exposure to hypoxic condition, mitochondria of either MSCs^rGDF11^ were densely populated and maintained elongated tubular morphology, whereas those from control MSCs were roundly shaped (Figure 3A,B). Furthermore, MSCs^si‐GDF11^ showed aggravated mitochondrial fragmentation (Figure S6). These data indicate that GDF11 attenuated mitochondrial fragmentation.

To determine the effect of GDF11 on mitochondrial functions of MSCs, we examined OCR (Figure 3C,D), intracellular ATP (Figure 3E) and mitochondrial membrane potential (Figure 3F). Similarly, there was no significant difference at OCR and ATP production between control MSCs and MSCs^rGDF11^ under normoxic condition (Figure 3C,E). When cultured at hypoxic condition, MSCs^rGDF11^ showed significantly higher OCR in both basal and maximal levels as compared with control MSCs (Figure 3D). Both ATP production (Figure 3E) and mitochondrial membrane potential (Figure 3F) were greater in MSCs^rGDF11^ than those in control MSCs. Knockdown of GDF11 resulted in a reversal of these effects (Figure S7).

### 3.4 Protective effect of GDF11 on mitochondrial morphology depends on OPA1

**FIGURE 4:**
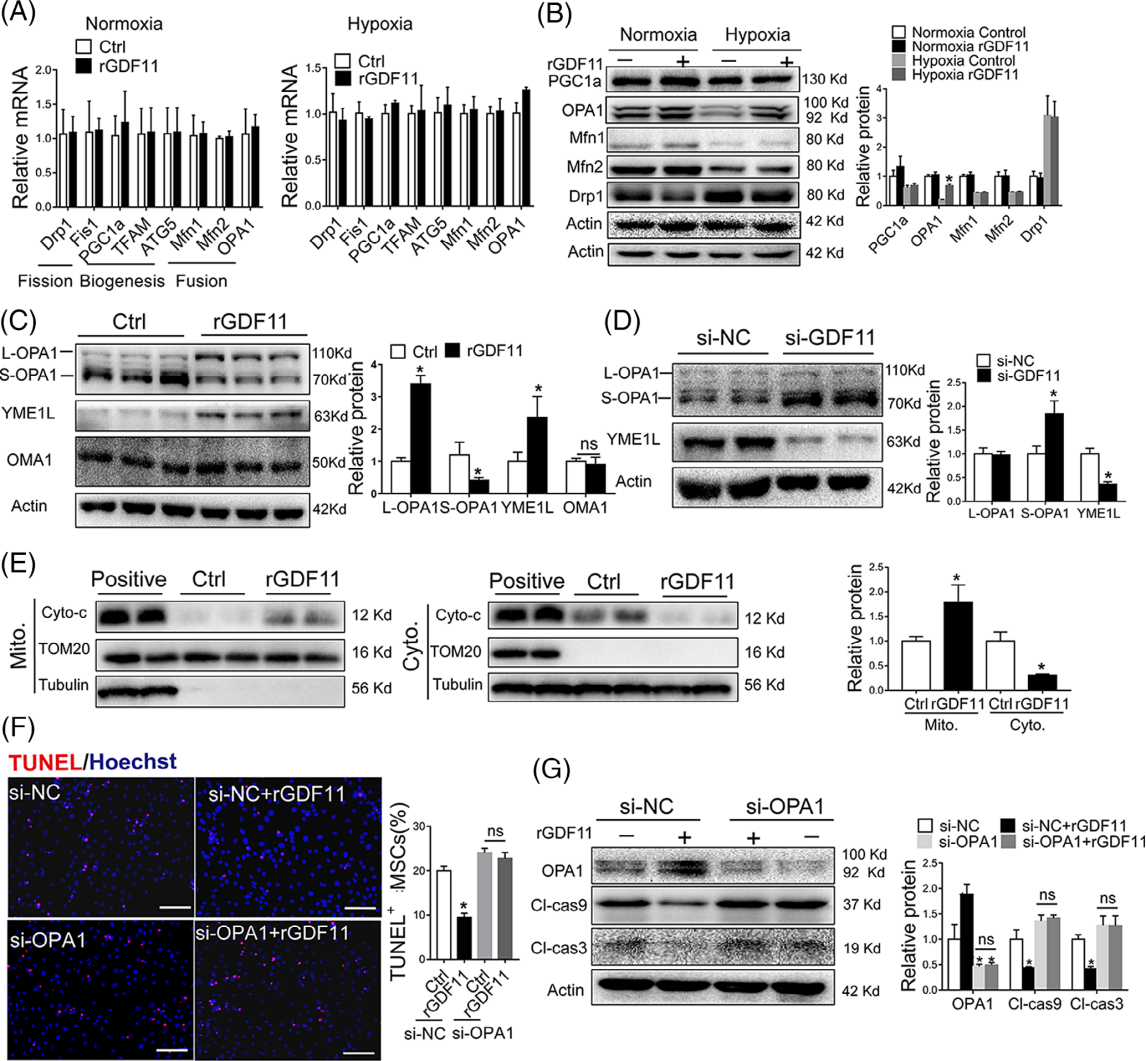
OPA1 was indispensable for the protective effects of GDF11 in response to hypoxic condition. A, Quantitative analysis of mRNAs of genes involved in mitochondrial homeostasis for MSCs^Ctrl^ and MSCs^rGDF11^ under normoxic or hypoxic condition. Relative mRNA levels were all compared with 18 seconds RNA. B, Western blot analysis of mitochondrial proteins: PGC‐1α, OPA1, Mfn1/2 and DRP1. Monoclonal antibody for OPA1 was used. Quantification of the proteins relative to control β‐actin was shown in right (n = 3). C, Western blot analysis of OPA1, YME1L and OMA1 in MSCs^Ctrl^ and MSCs^rGDF11^ under hypoxic condition. OPA1 detected by a polyclonal antibody. Quantification of the proteins relative to control β‐actin was shown in the bar graph (n = 3). D, Western blot analysis of OPA1 and YME1L in MSCs^si‐NC^ and MSCs^si‐GDF11^ in the same way as in C (n = 4). E, Western blot analysis of Cyto‐C in cytoplasm and mitochondrial. The values of Cyto‐C in mitochondrial were normalized with TOM20. And the values of Cyto‐C in cytoplasm were normalized with tubulin. Quantification was shown in the bar graph (n = 4). F, TUNEL staining of MSCs that were transfected with siRNAs (si‐NC and si‐OPA1), followed by treatment with rGDF11 for 24 hours and then exposed to hypoxia condition for 48 hours. Scale bar = 50 μm. Apoptotic cells were quantified by counting TUNEL‐positive nuclei out of total cells (n = 9). G, Western blot analysis of OPA1, cleaved caspase 3 and 9. Quantification of the proteins relative to control β‐actin was shown in the bar graph (n = 3). Data were shown as mean ± SD. *P < .05 vs si‐NC/Ctrl

To further investigate how GDF11 regulates mitochondrial dynamics, we analyzed the expression of proteins related to mitochondrial dynamics: OPA1 and Mitofusin1/2 (Mfn1/2) that are responsible for mitochondrial fusion, dynamin‐related protein 1 (Drp1) and mitochondrial fission 1 (Fis1) for fission, and peroxisome proliferator‐activated receptor‐γ coactivator‐1 alpha (PGC1α), mitochondrial transcription factor A (TFAM) and activating transcription factor 5 (ATF5) for biogenesis. There were no significant differences in mRNAs for these proteins between MSCs^Ctrl^ and MSCs^rGDF11^ at either normoxic or hypoxic conditions (Figure 4A), indicating that GDF11 did not affect the expression of mitochondrial dynamics‐related regulators at transcriptional level. Although no significant differences were detected for PGC1α, Mfn1, Mfn2, and Drp1 between MSCs^Ctrl^ and MSCs^rGDF11^, OPA1 protein was greatly increased under hypoxic condition in response to GDF11 treatment (Figure 4B). Furthermore, hypoxia stress induced decreased L‐OPA1 and increased S‐OPA1 expression levels in MSCs (Figure S8), which can be reversed by rGDF11 treatment of MSCs (Figure 4C). Similarly, this effect was attenuated by siRNA‐mediated knockdown of GDF11 (Figure 4D).

Previous studies demonstrated that the antiapoptotic effects of OPA1 were attributed to its role in regulating mitochondrial cristae morphology and cytochrome C distribution. In our present study, rGDF11 treatment resulted in better‐organized cristae dispersed within MSCs under hypoxia condition as compared with those without rGDF11 treatment (Figure S9A). Knockdown of GDF11 in MSCs resulted in loss of most of cristae membranes (Figure S9B). By separation of cytoplasmic and mitochondrial compartments, we found more cytochrome C in mitochondria and less in cytoplasm in MSCs^rGDF11^ than that in MSCs^Ctrl^ under hypoxia condition (Figure 4E), indicating that rGDF11 assisted the preservation of cytochrome C in mitochondria.

**FIGURE 5:**
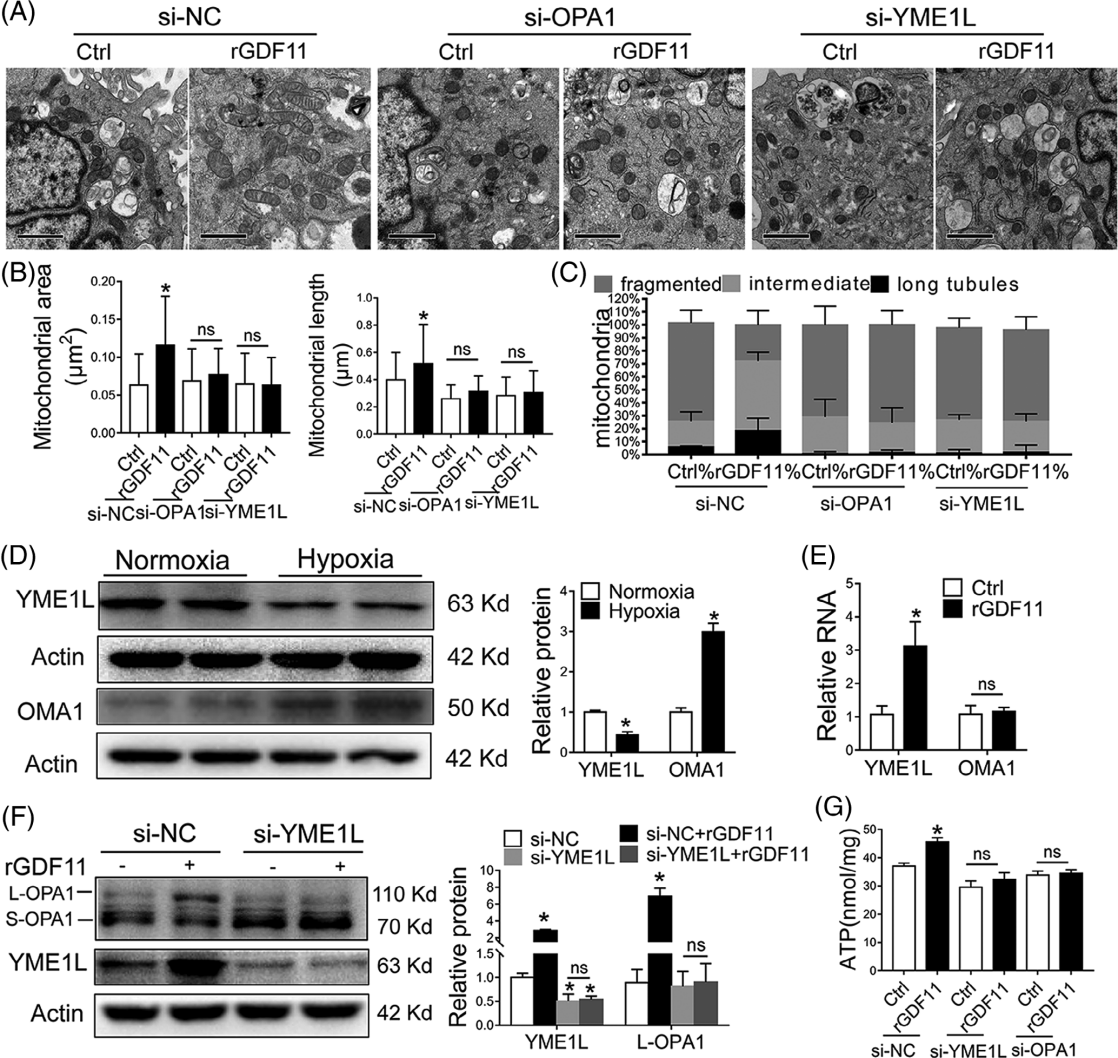
GDF11‐mediated OPA1 processing depends on YME1L. A, Representative TEM images of MSCs transfected with siRNA‐OPA1 or siRNA‐YME1L for 48 hours and then incubated with rGDF11 (50 ng/mL) for 24 hours and then exposed to hypoxia conditions for 48 hours (×10 000). Scale bars: 1 μm. B, C, Quantification of mitochondrial area, longitudinal length and size distribution according to their length: long tubules (>0.65 μm), intermediate (≤0.65 μm, ≥0.32 μm), and fragmented (<0.32 μm) (n = 105 for MSC^si‐NC^, n = 64 for MSCs^si‐NC + rGDF11^, n = 123 for MSC^si‐OPA1^, n = 68 for MSC^si‐OPA1 + rGDF11^, n = 93 for MSC^si‐YME1L^, and n = 96 for MSC^si‐YME1L + rGDF11^). D, Representative immunoblots and densitometric quantification for the expression of YME1L and OMA1 under normoxic and hypoxic conditions (n = 4). E, mRNA levels of YME1L and OMA1 in MSCs^Ctrl^ and MSCs^rGDF11^, and 18 seconds served as control. F, Western blot analysis of YME1L and OPA1. YME1L and L‐OPA1 were quantified and presented as relative level by comparing with β‐actin control (n = 3). G, Intracellular ATP levels in MSCs at specified conditions were determined. ATP levels was calibrated with protein content (n = 3). Data were shown as mean ± SD. *P < .05 vs si‐NC

To assess whether the integrity of OPA1 is indispensable for the GDF11‐mediated MSCs survival, we knocked down OPA1 expression by using siRNA specific for OPA1 (si‐OPA1) while using scrambled siRNA (si‐NC) as a control (Figure S2C). There was no significant difference in MSCs apoptosis (Figure 4F) and levels of cleaved caspase‐3 and caspase‐9 (Figure 4G) between MSCs^Ctrl^ and MSCs^rGDF11^ when OPA1 was knocked down, indicating that effect of rGDF11 on protecting MSCs from apoptosis is OPA1‐dependent. Furthermore, after OPA1 in MSCs was silenced by siRNA, the effects of rGDF11 on preventing mitochondrial fragmentation and loss of cristae (Figure 5A‐C and Figure S10), mitochondrial membrane potential (Figure S11A,B), and intracellular ATP production content (Figure 5E) were all diminished, indicating that protective effects of GDF11 on mitochondria under hypoxia condition also mainly depend on OPA1.

### 3.5 GDF11 modulated OPA1 processing through upregulation of YME1L

OPA1 is proteolytically processed by mitochondrial proteases OMA1 and YME1L to generate short forms^20^, of that YME1L accelerates the mitochondrial fusion and OMA1 mediates mitochondrial fragmentation.^14^, ^21^, ^22^ In our study, we observed more OMA1 and less YME1L in MSCs under hypoxic condition (Figure 5D). Treatment of MSCs with rGDF11 increased YME1L expression in MSCs under hypoxia condition, but did not affect OMA1 expression (Figure 4C, Figure 5E). Knockdown of GDF11 by siRNA inhibited the expression of YME1L under hypoxic condition (Figure 4D). Downregulation of YME1L by siRNA diminished GDF11‐induced upregulation of L‐OPA1 (Figure 5F). Furthermore, knockdown of YME1L blocked GDF11‐mediated reduction of apoptosis and downregulation of cleaved caspase 3 and 9 (Figure S11C,D), diminished the favorable effects of rGDF11 on characteristics of mitochondrial morphology, resulting in mitochondrial fragmentation and cristae vanished (Figure 5A‐C Figure S10). Being consistent with the changes in morphology, the functions of mitochondria, including mitochondrial membrane potential (Figure S11A,B) and ATP production (Figure 5G), were deteriorated in MSCs after being transfected with siRNA‐YME1L, either with or without rGDF11. Taken together, the results strongly supported that GDF11 promotes mitochondrial fusion to alleviate apoptosis of MSCs under hypoxic condition in an YME1L‐mediated OPA1 bioprocessing.

### 3.6 Effect of GDF11 on mitochondria was through ALK5‐Smad2/3 signaling pathway

**FIGURE 6:**
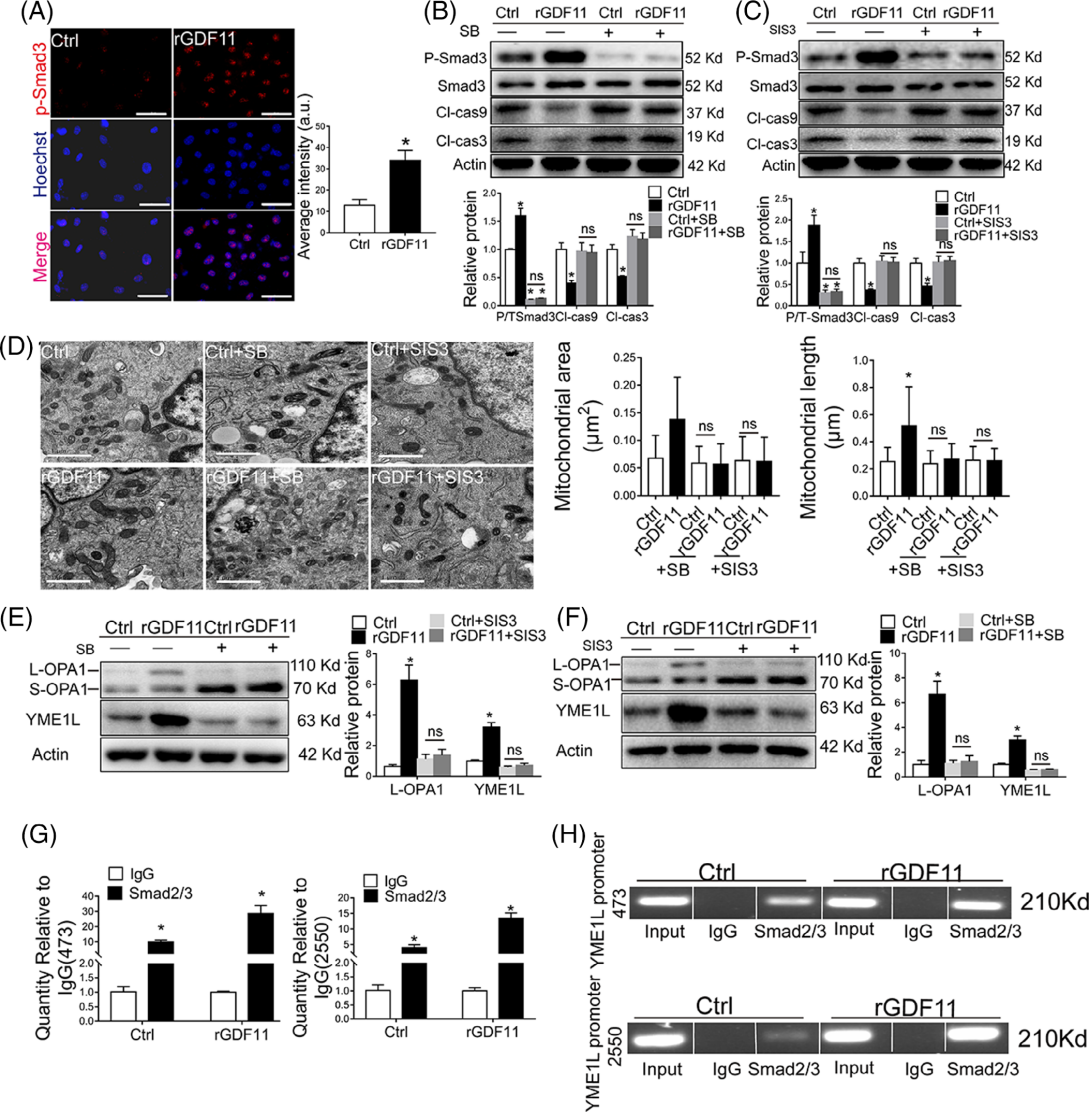
GDF11 protected MSCs from hypoxia‐induced apoptosis through ALK5‐Smad2/3 pathway. A, Localization of p‐Smad3 in MSCs. DAPI were stained with for nuclear (blue) and fluorescence‐labeled Ab against phosphorylated Smad3 (red). Scale bar = 50 μm. Fluorescence intensity was quantified. B, C, Western blot analysis of p‐Smad3, cleaved caspase 3 and 9 in MSCs which were treated with TGFβR1 inhibitor SB4431542 (B) or p‐Smad3 inhibitor SIS3 (C) for 30 minutes, and incubated with rGDF11 (50 ng/mL) for 24 hours and then exposed to hypoxia condition for 48 hours (n = 3 in B and C). D, Representative TEM images of MSCs treated as described in (B, C) (×10 000). Scale bar =1 μm. Mitochondria were visually scored for their area and longitudinal length. n = 149 for MSC^Ctrl^, n = 63 for MSCs^rGDF11^, n = 153 for MSC^Ctrl + SB^, n = 160 for MSC^rGDF11 + SB^, n = 151 for MSC^Ctrl + SIS^ and n = 139 for MSC ^rGDF11 + SIS^. E, F, Immunoblot analysis of L‐OPA1 and YME1L in MSCs treated as specified. Relative proteins were presented by comparing each band density with that of β‐actin (n = 3 in E and F). G, H, The abilities of Smad2/3 binding to YME1L promoter sites at 473 to 485 and 2550 to 2562 were analyzed by ChIP assay. The purified DNA and input genomic DNA were analyzed by real‐time PCR (G). The PCR products were analyzed by gel electrophoresis (H). Each in vitro experiment was repeated three times. Data were shown as mean ± SD. *P < .05 vs IgG, ^#^ *P <* .05 vs Ctrl

To investigate underlying mechanism of GDF11‐mediated upregulation of YME1L, canonic TGF‐β receptor/Smad2/3 pathway was examined. We found that treatment of MSCs with rGDF11 significantly increased the expression of TGF‐β type I receptor ALK5 (Figure S12) and phosphorylation of Smad2/3 (Figure 6A‐C), while no significant difference was observed for other TGF‐β receptors (ALK4 and ALK7 or ActRIIA and ActRIIB) (Figure S12). Treatment of MSCs with ALK5 inhibitor SB431542 or Smad3 inhibitor SIS3 blocked the effect of rGDF11 on phosphorylation of Smad3 and on apoptosis‐related cleaved caspase 3/9 (Figure 6B,C), and suppressed GDF11‐induced antiapoptosis effect on MSCs under hypoxic condition (Figure S13A).

Furthermore, SB431542 or SIS3 inhibited the rGDF11‐mediated effects on mitochondria morphology (Figure 6D, Figure S13B), mitochondria membrane potential (Figure S14A,B) and ATP production (Figure S14C). Either SB431542 or SIS3 also diminished GDF11‐mediated upregulation of YME1L and L‐OPA1 (Figure 6E,F). Enhancement of tube formation of HUVECs by the conditioned medium of MSCs pretreated with rGDF11 was also eliminated when MSCs were cultured in the presence of SB431542 or SIS3 (Figure S15). These results indicated that ALK5‐Smad2/3 signaling pathway was essential for GDF11‐mediated mitochondrial protection in MSCs.

### 3.7 Smad2/3 regulated YME1L transcription by binding to 3′ UTR of YME1L promoter

Previous studies have shown that phosphorylated Smad2/3 translocated into nucleus and functioned as a transcriptional factor.^23^ To determine whether Smad2/3 directly regulated YME1L expression, the promoter of YME1L was analyzed using the Jaspar software, and two putative binding sequences for Smad2/3 (sites 473‐485 and 2550‐2562) were identified (Table S2), which implied that Smad2/3 could enhance YME1L transcription by directly binding to the 3′ untranslated regions (UTR) of YME1L promoter. This was proved by ChIP‐qPCR assay which also showed that recruitment of Smad2/3 to YME1L promoter at sites 473 to 485 and 2550 to 2562 could be enhanced by rGDF11 in MSCs under hypoxic condition (Figure 6G,H), confirming that GDF11‐induced effects in MSCs is through upregulation of YME1L by direct binding of Smad2/3 to its promoter.

### 3.8 Delivery of MSCs^LV‐GDF11^ resulted in better cardiac function after MI

**FIGURE 7:**
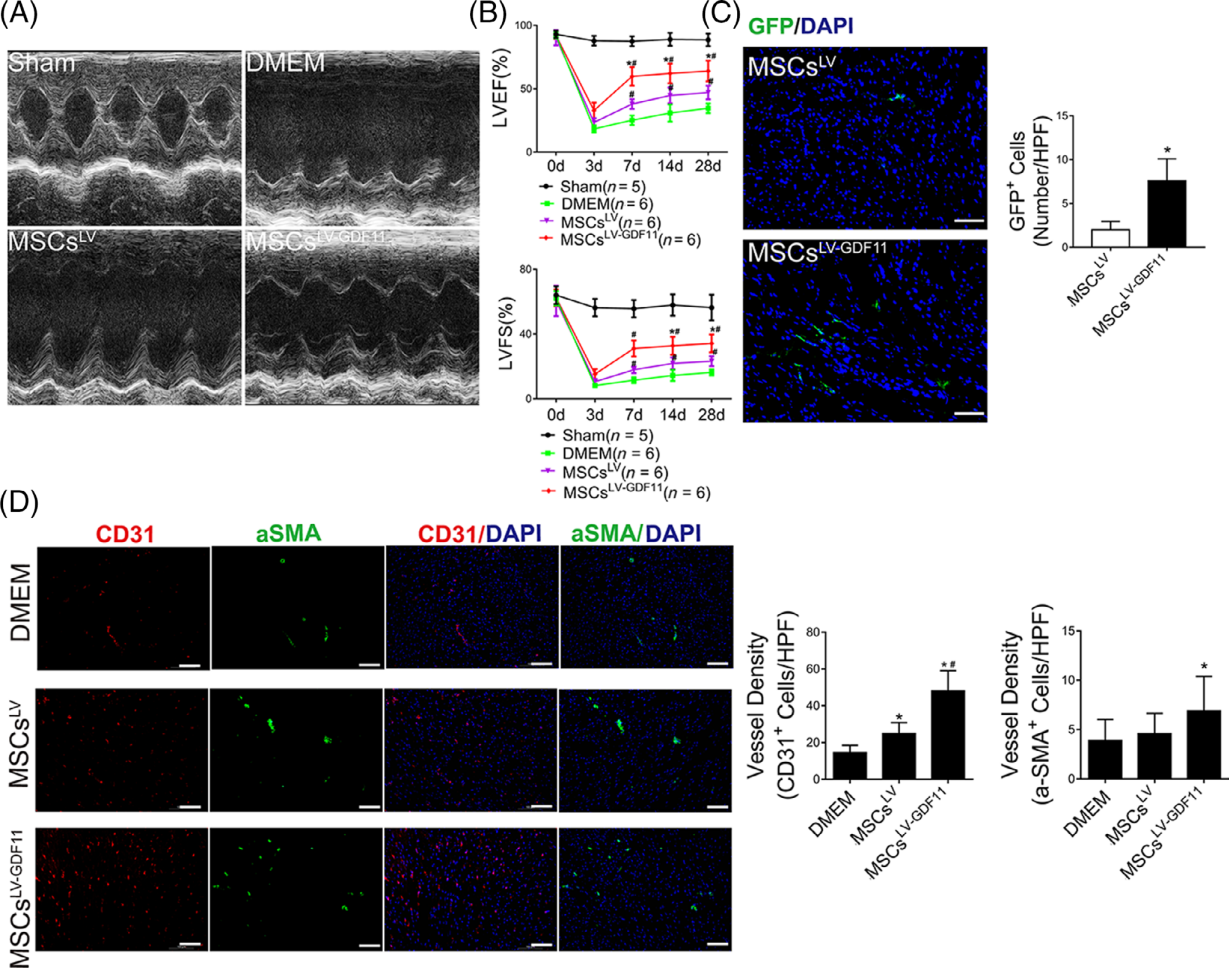
Delivery of GDF11 overexpressing MSCs improved cardiac function after MI in vivo. A, Representative echocardiographic M‐mode images showed the changes of cardiac function in each group at 28 days after MI. B, Left ventricular ejection fraction and left ventricular fractional shortening were quantified at 3, 7, 14, and 28 days post MI (n = 5 for Sham group, n = 6 for DMEM group, n = 6 for MSCs^LV^ group, and n = 6 for MSCs^LV‐GDF11^ group, respectively). C, MSCs retention at day 3 post‐MI (n = 6 for each group). Representative images of GFP staining were assayed by fluorescence microscopy, wherein GFP‐positive cells (in green) were specifically counted for MSCs retention, with nuclei stained with DAPI. Scale bar = 100 μm. D, Representative immunofluorescence image of CD31 staining for capillaries (red) and α‐SMA staining for arterioles (green) with nuclei stained with DAPI in the remote zone of ischemic heart from DMEM, MSCs^LV^, and MSCs^LV‐GDF11^ group mice at day 28 post‐MI. The capillary and arteriolar densities in the remote zones are quantified by 6‐8 HPFs per section in the bar graphs. Scale bar =100 μm. Data are shown as mean ± SD. *P < .05 vs DMEM/MSCs^LV^, ^#^ *P <* .05 vs MSCs^LV^

To validate the therapeutic effects of MSCs^LV‐GDF11^ in cardiac regeneration, equal numbers of either MSCs^LV‐GDF11^ or MSCs^LV^ were injected into the peri‐infarct zone of mouse heart immediately after MI. Treatment with MSCs^LV‐GDF11^ resulted in a better recovery of cardiac function in comparison with that with MSCs^LV^ or DMEM (Figure 7A,B). Increased numbers of GFP‐positive cells (Figure 7C) and lower numbers of apoptotic cells in the peri‐infarct area (Figure S16A) were detected in MSCs^LV‐GDF11^ group as compared with MSCs^LV^ group at day 3 post‐MI, indicating that better MSC retention and stronger antiapoptotic effect were achieved when MSCs overexpressing GDF11. These were concomitant with reduced scar size (Figure S16B) and more angiogenesis in aspect of increased number of ECs and smooth muscle cells in the remote area at day 28 post‐MI (Figure 7D). All these results support that GDF11 enhanced therapeutic effects of MSCs.

## 4 DISCUSSION

In this study, we found a novel role of GDF11 in enhancing the paracrine function of cardiac MSCs and protecting MSCs from apoptosis under hypoxic condition. We verified that GDF11 conferred the protective effect of MSCs by preserving mitochondrial function and promoting their fusion through its binding with TGF‐β receptor and activating Smad2/3/YME1L/OPA1 signaling pathway. Two Smad2/3 binding sites at YME1L promoter were discovered, and GDF11‐mediated activation of Smad2/3 promoted the expression of YME1L by direct binding with its promoter. Transplantation of MSCs overexpressing GDF11 into infarct heart resulted in better MSCs survival and retention, leading to more angiogenesis, smaller scar size, and improved cardiac function in comparison with the control MSCs. Our results provided a novel approach to potentiate therapeutic efficacy of stem cells for cardiovascular indications.

MI induces an irreversible loss of cardiomyocytes and scar formation, and may ultimately result in heart failure. Stem cell therapy is a promising strategy for treatment of heart failure after MI, in which MSCs are the most used cell type due to their diverse inherent properties, such as low immunogenicity, multipotentiality, and maintenance of “stemness.”^17^, ^24^, ^25^ MSCs can be derived from different tissues, for example, bone marrow, perinatal tissues, endometrium, and adipose tissues. Since the preclinical investigations support the concept that donor cells more oriented toward a cardiovascular phenotype favor better repair,^26^ we used cardiac MSCs from heart to study the therapeutic effect of MSCs at different levels of GDF11. Cardiac MSCs possess mesenchymal stromal characteristics with classic mesenchymal stromal markers (Figure S1). They have been showed playing a critical role in cardiac remodeling during pathological conditions.^26^, ^27^ Regardless of the origin of MSCs, low survival of transplanted MSCs in infarcted myocardium limits successful use of MSCs in treating MI. Significant attempts have been made to enhance cellular and therapeutic efficacy of MSCs by preconditioning.^25^, ^28^

As a member of TGF‐β superfamily, GDF11 not only contributes to embryonic development and histogenesis but also plays a role in metabolic disorders, cancers, and heart‐ and skeletal‐muscle rejuvenation in aged mice.^29^ Numerous studies reported positive effects of GDF11 in cell migration, proliferation, tube formation, and adherence.^2^, ^30^, ^31^ In our study, we found that GDF11 significantly improved not only viability of MSCs under hypoxic condition (Figure 1, Figure S3B) but also the paracrine effect of MSCs (Figure 2). For in vivo, injection of GDF11‐overexpressing MSCs had better cell retention, and resulted in better cardiac function, angiogenesis and decreased cardiac fibrosis in a mouse MI model (Figure 7). However, some controversies about GDF11 were reported. For example, studies showed that GDF11 suppressed migration of neural stem cells,^32^ while others showed that GDF11 increases proliferation of ECs from brain‐capillary and migration of endothelial progenitor cells.^31^, ^33^ These results suggest that the role of GDF11 could be tissue‐specific.

Enhanced paracrine effects of MSCs by GDF11 could be the accumulated results of both better retention/survival and more production of growth factors and cytokines from MSCs, for example, VEGFA (Figure 2B‐C). It has been reported that TGF‐β signaling activates protein kinase A (PKA),^34^ and PKA can stimulate angiogenesis by inducing VEGF expression in ECs.^35^ We have also found that expression of VEGFA was similarly enhanced by GDF11 through PKA signaling pathway in cardiomyocytes (data not shown). We speculate that GDF11 may also upregulate VEGF expression in MSCs in a similar way.

Maintenance of mitochondrial integrity has a vital role in cellular resistance to apoptosis. Mitochondrial integrity such as morphology, number, and size is controlled through a series of processes, including mitochondrial biogenesis, mitochondrial dynamics, mitophagy, and transport processes.^7^ Under normoxic condition, mitochondria are balanced between fusion and fission. We observed no significant difference in the morphology of mitochondria between control and rGDF11‐treated MSCs (Figure S5), indicating that GDF11 has no effect on the homeostasis of mitochondria at normal condition. Under pathological condition, mitochondrial dysfunction results in activation of mitochondrial fission and inactivation of mitochondrial fusion.^7^, ^36^ In the present study, we confirmed that mitochondria in MSCs under hypoxia condition were fragmented, along with lower ATP production, and inhibition of respiratory chain (Figure 3). These changes lead to mitochondrial swelling that damages the mitochondrial ultrastructure and results in release of cytochrome c and apoptosis‐related markers into the cytoplasm (Figure 4E). When GDF11 was given externally in the culture of MSCs, mitochondria fission is inhibited and fusion is enhanced, more elongated tubular mitochondrial networks and better cristae in MSCs were observed, in association with less apoptotic cells (Figure 3A,B).

Previous studies have shown that GDF11 can induce activation of PGC‐1α, which controls expression of many mitochondrial proteins,^37^ indicating that GDF11 could involve in mitochondrial quality control by regulating mitochondrial biogenesis or mitochondrial dynamics. Attenuated mitochondrial fragmentation, well‐organized cristae, enhanced respiratory capacity, upregulation of ATP production, increased mitochondrial membrane potential were detected in MSCs pretreated with rGDF11 under hypoxic condition, and knockdown of GDF11 led to an opposite result (Figures 3 and 4). Intact and tighten cristae can preserve cytochrome c in intermembrane space and inhibit its release in response to hypoxia. In our study, GDF11 can alleviate cytochrome c release. The cytochrome c released from mitochondria to cytosol initiate the activation of caspase‐3 and caspase‐9.^38^ Consistent with this, our study revealed that GDF11 could enhance survival of MSCs under hypoxic condition through regulation of mitochondrial dynamics.

The regulation of mitochondrial dynamics by GTPase OPA1, which is located at the inner mitochondrial membrane, is crucial for adapting mitochondrial function and maintaining cristae structure.^39^ Loss of OPA1 leads to mitochondrial fragmentation,^20^ which consists with our finding (Figure 5A‐C). Embryonic fibroblasts in OPA1 knockout mouse exhibit increased cell death.^40^ Proteolytic processing of OPA1 is a critical regulatory step to balance mitochondrial fusion/fission in cardiomyocytes.^9^, ^20^, ^41^ L‐OPA1 is associated with fusion, whereas S‐OPA1 leads to fission.^42^ Indeed, we observed that under hypoxia condition, more L‐OPA1 was converted into short isoforms (Figure S8), mitochondria were fragmented and cellular functions were deteriorated. Culturing with external GDF11, L‐OPA1 in MSCs were significantly increased. Knocking down OPA1 blocked the effect of GDF11 on the morphological dysfunction and apoptosis (Figure S7). The data suggest that GDF11 regulates OPA1 processing in MSCs under hypoxic condition.

It is known that OPA1 is proteolytically processed by the mitochondrial proteases OMA1 and YME1L. S‐OPA1 is formed by cleavage at S1 and S2 sites of L‐OPA1 by OMA1 and YME1L, respectively.^21^ Maintaining a proper ratio between L‐ and S‐OPA1 is required for maintenance of mitochondrial morphology.^43^ It has been reported that OMA1 can be degraded by YME1L in depolarized mitochondria containing high levels of ATP.^44^ However, we did not observe significant change in OMA1 expression in MSCs at hypoxia condition with or without GDF11, whereas GDF11 significantly increased YME1L expression upon hypoxia stimulation (Figure S11A,B). Previous study showed that knockout of OMA1 resulted in more L‐OPA1,^21^ which agrees with our observation that a higher ratio of L/S‐OPA1 was maintained when GDF11 induced more YME1L expression (Figure 4C). The increased YME1L may competitively inhibit proteolysis of L‐OPA1 by OMA1, therefore enhanced mitochondrial fusion. Knockdown of YME1L in MSCs inhibited GDF11‐induced increased L‐OPA1 (Figure 5F), confirming that GDF11‐induced OPA1 cleavage depends on YME1L. In addition, the YME1L and OPA1 expression levels in MSCs were decreased after the deletion of GDF11 (Figure 4D). As expected, the effect of GDF11 on mitochondrial morphology vanished after knockdown of YME1L expression (Figure 5A‐C). All these data confirmed that GDF11 acts through YME1L‐mediated OPA1 process. Upregulated YME1L expression was responsible for rebalanced OPA1 processing in MSCs, and leads to restoration of mitochondrial morphology as well as better mitochondria function under hypoxia condition.

GDF11 binds to TGF‐β type II receptors, which recruits activin type I receptors to activate the Smad2/3 pathway associated with the common mediator Smad4.^45^ TGF‐β activin type I receptors include ALK4, ALK5, and ALK7. Inhibition of TGF‐β type I receptor and Smad2/3 using specific inhibitors abrogate effect of GDF11 on upregulating YME1L and L‐OPA1 (Figure 6E,F). Bioinformatic analysis predicted that YME1L has two potential response elements for SMAD2/3/4 in its promoter region. We used ChIP‐qPCR assay to confirm that GDF11 promoted the recruitment of p‐Smad2/3 to the promoter of YME1L in MSCs under hypoxic condition (Figure 6G). These results unveiled that GDF11 upregulated YME1L expression to modulate OPA1 cleavage processing through ALK5‐Smad2/3 pathway, thus proving mitochondrial protection in MSCs. Besides the canonical TGF‐β/Smad2/3 signaling pathway, GDF11 can also activate several noncanonical signaling pathways in a context‐dependent manner including AKT, AMPK, and ERK1/2 pathway.^3^, ^31^, ^46^ It needs further study to determine if the above‐mentioned nonclassical pathways also involve in the modulation of mitochondrial morphological functions by GDF11.

Taken together, our results demonstrate that GDF11 exerted its protective effect on MSCs through maintaining mitochondrial morphology and activity and preventing apoptosis under hypoxia condition. Such an effect was achieved via ALK5‐Smad2/3 dependent pathway, enhancing the binding of phosphorylated Smad2/3 onto the promoter of YME1L, resulting upregulation of YME1L and conserved OPA1 cleavage process to inhibit fission and to promote fusion of mitochondria (Figure S17).

## 5 CONCLUSION

This study, for the first time, demonstrated a crucial role of GDF11 on mitochondrial integrity to protect stem cells in ischemic environments. GDF11 improved the activity of MSCs through rebalancing OPA1 processing and promoting mitochondrial fusion. Our study provided potential targets to improve clinical application of stem cell therapy to treat cardiovascular diseases by maintaining mitochondrial dynamic homeostasis.

## CONFLICT OF INTEREST

The authors declared no potential conflicts of interest.

## AUTHOR CONTRIBUTIONS

Y.Z., J.Z.: conception and design, experiments conduction, data analysis and manuscript writing; N.Z., Q.L., Y.W.: experiments conduction and data analysis; X.H., W.Z., J.C.: final approval of manuscript; H.Y.: conception and design, final approval of manuscript.

## Supporting information


**Figure S1.** Supporting information

**Figure S2.** Supporting information

**Figure S3.** Supporting information

**Figure S4.** Supporting information

**Figure S5.** Supporting information

**Figure S6.** Supporting information

**Figure S7.** Supporting information

**Figure S8.** Supporting information

**Figure S9.** Supporting information

**Figure S10.** Supporting information

**Figure S11.** Supporting information

**Figure S12.** Supporting information

**Figure S13.** Supporting information

**Figure S14.** Supporting information

**Figure S15.** Supporting information

**Figure S16.** Supporting information

**Figure S17.** Supporting information

**Data S1.** Supporting information

**Table S1.** Sequences of primers and siRNAs used in this study

**Table S2.** YME1L promoter sequences that were predicted to be bound by SMAD2/3.

## Data Availability

The data that support the findings of this study are available from the corresponding author upon reasonable request.
