# Supplementary material for: GDF11 enhances therapeutic efficacy of mesenchymal stem cells for myocardial infarction via YME1L‐mediated OPA1 processing
Source: Stem Cells Transl Med. 2020 Jun 9;9(10):1257–71. doi: 10.1002/sctm.20-0005 (PMC7519765; doi:10.1002/sctm.20-0005)
Supplement: Supplementary file 1 — Figure S1. Supporting information [file SCT3-9-1257-s011.pdf]

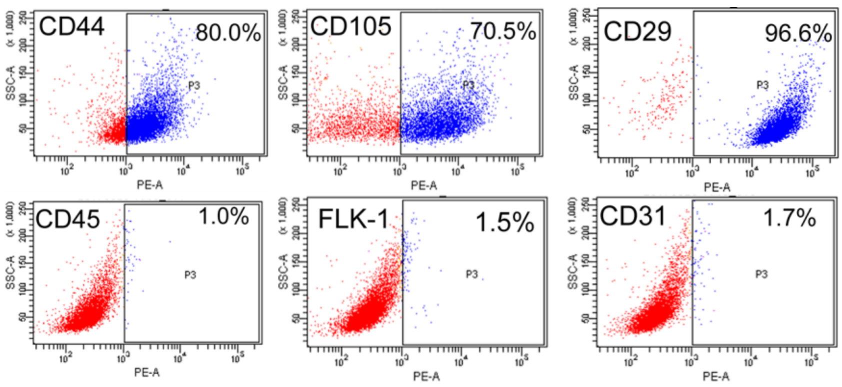

Figure. S1 Characteristics and identification of MSCs derived from mouse heart. MSCs were identified with the positive cell surface markers CD44, CD105, and CD29, and negative for the CD45(hematopoietic surface marker) and Flk-1and CD31 (endothelial cells surface marker) by flow cytometry.
