# Supplementary material for: GDF11 enhances therapeutic efficacy of mesenchymal stem cells for myocardial infarction via YME1L‐mediated OPA1 processing
Source: Stem Cells Transl Med. 2020 Jun 9;9(10):1257–71. doi: 10.1002/sctm.20-0005 (PMC7519765; doi:10.1002/sctm.20-0005)
Supplement: Supplementary file 2 — Figure S2. Supporting information [file SCT3-9-1257-s013.pdf]

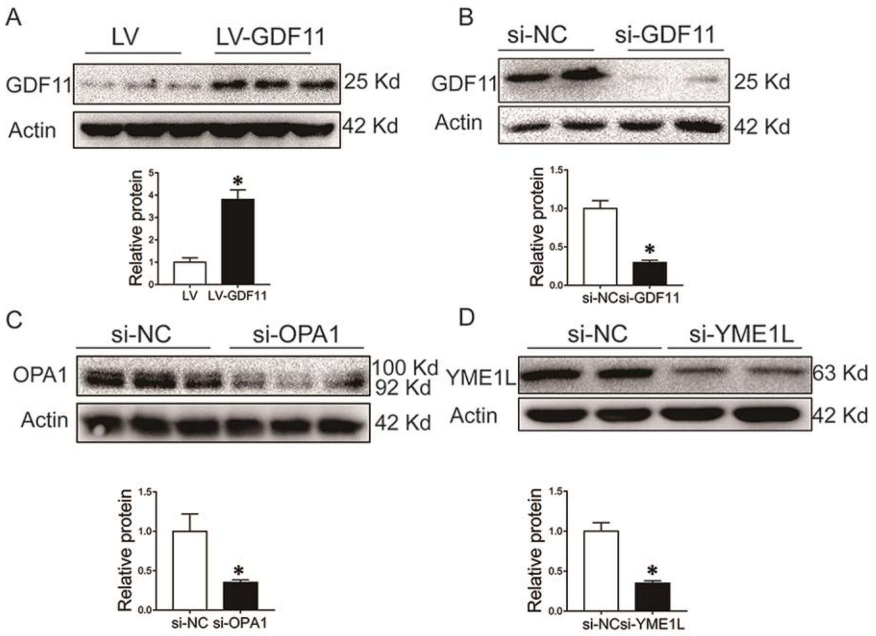

Figure. S2 Quantification of gene expression in MSCs by western blot. **A.** Representative immunoblots and densitometric quantification for GDF11 overexpression efficiency in MSCs transfected with lentivirus or lenti-GDF11 (n=3). **B.** Representative immunoblots and densitometric quantification for GDF11 knockdown efficiency in MSCs transfected with siRNA-NC or siRNA-GDF11 (n=4). **C.** Representative immunoblots and densitometric quantification for OPA1 knockdown efficiency in MSCs transfected with siRNA-NC and siRNA-OPA1 (n=3). **D.** Representative immunoblots and densitometric quantification for YME1L knockdown efficiency in MSCs transfected with siRNA-NC and siRNA-YME1L (n=4). β-Actin served as control. Data were shown as mean ± SD. * *P<0.0*5 vs siRNA-NC/LV.
