# Supplementary material for: GDF11 enhances therapeutic efficacy of mesenchymal stem cells for myocardial infarction via YME1L‐mediated OPA1 processing
Source: Stem Cells Transl Med. 2020 Jun 9;9(10):1257–71. doi: 10.1002/sctm.20-0005 (PMC7519765; doi:10.1002/sctm.20-0005)
Supplement: Supplementary file 3 — Figure S3. Supporting information [file SCT3-9-1257-s014.pdf]

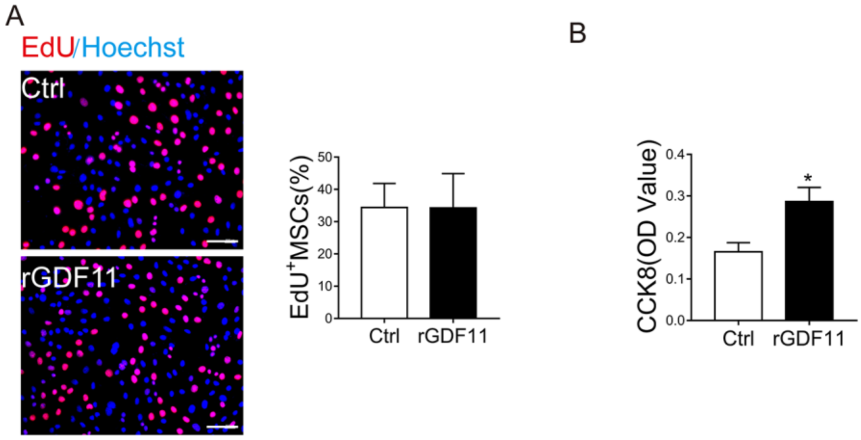

Figure. S3 GDF11 has no effect on proliferation of MSCs but improved cell survival under hypoxic condition. A. Representative EdU staining images of MSCs with rGDF11 (50ng/ml) for 24h under normoxic condition. Pink represents EdU positive cells. The nuclear of all cells were stained with Hochest showing blue. Scale bar =50μm. Quantification of proliferation cells by EdU-positive nuclei (n=9). B. Cell survival under hypoxic condition was detected by CCK8 assay. MSCs were pretreated with rGDF11(50ng/ml) for 24h and then exposed to hypoxia for 48h (n=4). Data were shown as mean ± SD. * *P<0.05* vs. Ctrl.
