# Supplementary material for: GDF11 enhances therapeutic efficacy of mesenchymal stem cells for myocardial infarction via YME1L‐mediated OPA1 processing
Source: Stem Cells Transl Med. 2020 Jun 9;9(10):1257–71. doi: 10.1002/sctm.20-0005 (PMC7519765; doi:10.1002/sctm.20-0005)
Supplement: Supplementary file 4 — Figure S4. Supporting information [file SCT3-9-1257-s015.pdf]

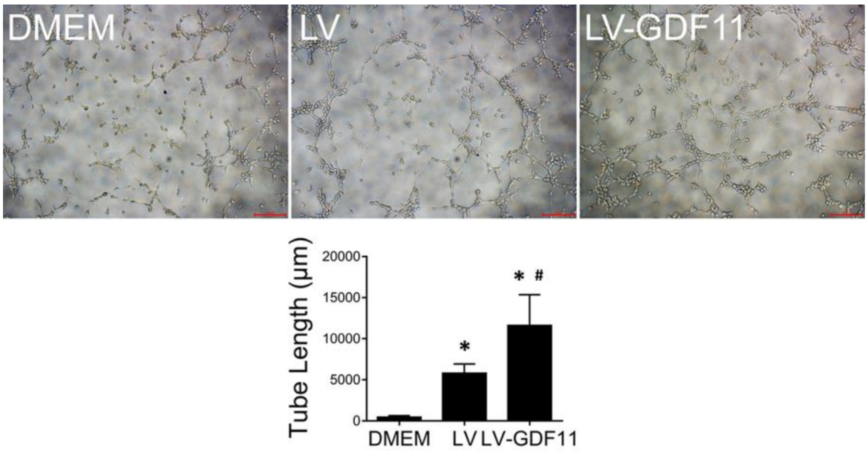

Figure. S4 Tube formation assay of HUVECs. Representative images of tube formation assay of HUVECs which were cultured with DMEM or with conditioned medium of MSCs that had been transduced with lentiviral vector carrying no gene (LV) or with GDF11 gene (LV-GDF11). Scale bar =100μm. Quantification of tube formation was shown in bar graphs (n=10). GDF11 enhanced paracrine effects of MSCs in vitro. Scale bar = 100μm. Data are shown as mean ±SD. *$P<0.05$ vs. DMEM, $^{\#}P<0.05$ vs LV.
