# Supplementary material for: GDF11 enhances therapeutic efficacy of mesenchymal stem cells for myocardial infarction via YME1L‐mediated OPA1 processing
Source: Stem Cells Transl Med. 2020 Jun 9;9(10):1257–71. doi: 10.1002/sctm.20-0005 (PMC7519765; doi:10.1002/sctm.20-0005)
Supplement: Supplementary file 5 — Figure S5. Supporting information [file SCT3-9-1257-s016.pdf]

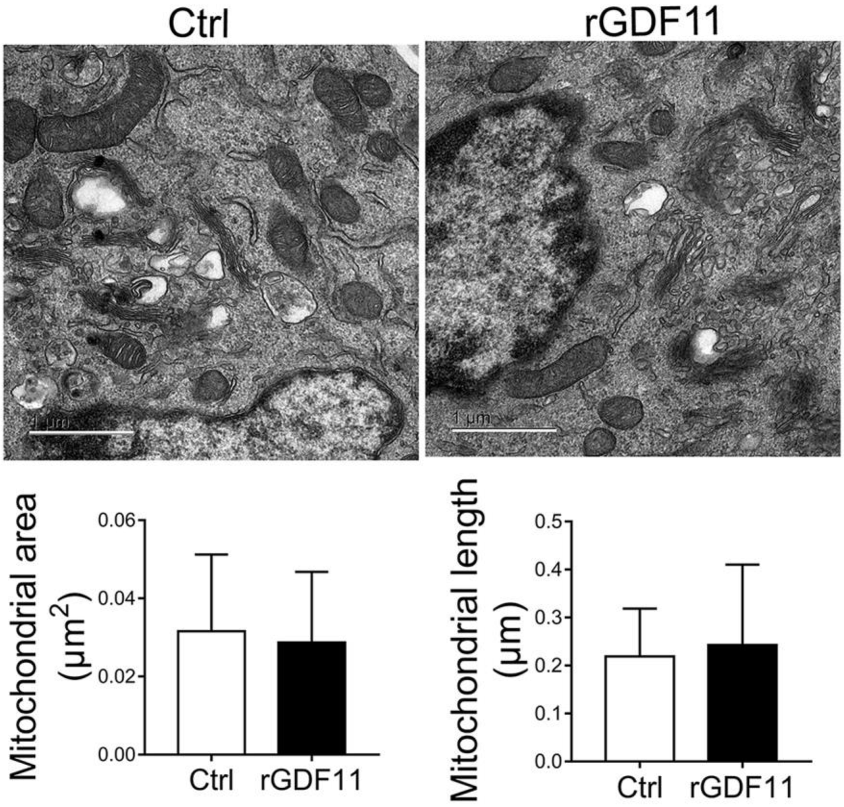

Figure. S5 Mitochondria morphology observed by TEM under normoxic condition. Representative images of MSCs pretreated with or without rGDF11 under normoxic conditions were shown by electron microscopy (magnification was set at ×10,000). Scale bar = 1μm. Quantification of mitochondrial area and longitudinal length was presented in bar graphs. Mitochondria were visually scored (n=44 for $MSCs^{Ctrl}$, and n=32 for $MSCs^{rGDF11}$). Data were shown as mean ± SD.
