# Supplementary material for: GDF11 enhances therapeutic efficacy of mesenchymal stem cells for myocardial infarction via YME1L‐mediated OPA1 processing
Source: Stem Cells Transl Med. 2020 Jun 9;9(10):1257–71. doi: 10.1002/sctm.20-0005 (PMC7519765; doi:10.1002/sctm.20-0005)
Supplement: Supplementary file 6 — Figure S6. Supporting information [file SCT3-9-1257-s017.pdf]

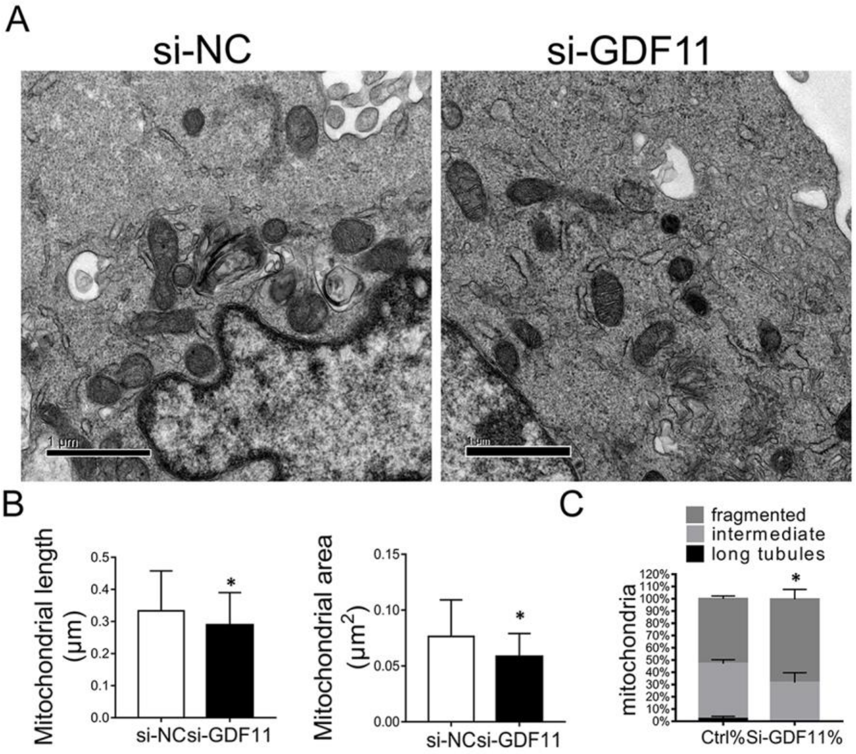

Figure. S6 Deficiency of GDF11 deteriorated mitochondrial morphology under hypoxic condition. **A.** Representative images of $MSCs^{si\text{-}NC}$ and $MSCs^{si\text{-}GDF11}$ under hypoxia condition were shown by electron microscopy (magnification was set at ×10000, respectively). Scale bar = 1μm. **B, C.** Quantification of mitochondrial area, longitudinal length and distribution of mitochondria according to their length: long tubules (> 0.65μm), intermediate (≤0.65μm, ≥0.32μm) and fragmented (< 0.32μm). Mitochondria were visually scored (n=105 for $MSCs^{si\text{-}NC}$ and n=70 for $MSCs^{si\text{-}GDF11}$). Three independent experiments were repeated. Data were shown as mean ± SD. *$P<0.05$ vs. si-NC.
