# Supplementary material for: GDF11 enhances therapeutic efficacy of mesenchymal stem cells for myocardial infarction via YME1L‐mediated OPA1 processing
Source: Stem Cells Transl Med. 2020 Jun 9;9(10):1257–71. doi: 10.1002/sctm.20-0005 (PMC7519765; doi:10.1002/sctm.20-0005)
Supplement: Supplementary file 7 — Figure S7. Supporting information [file SCT3-9-1257-s018.pdf]

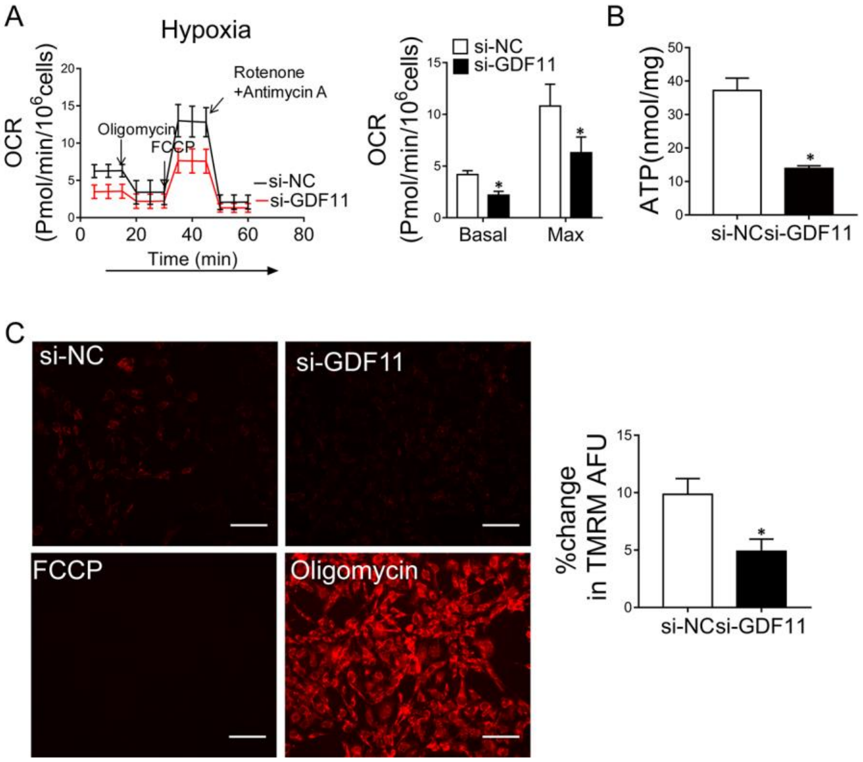

Figure. S7 Deficiency of GDF11 deteriorated mitochondrial function under hypoxic condition. **A.** Mitochondrial respiration reflected by Oxygen consumption rate (OCR) were detected in MSCs with si-NC and si-GDF11 under hypoxia conditions. **B.** ATP levels of MSCs with si-NC and si-GDF11. **c** Images of $MSCs^{si\text{-}NC}$ and $MSCs^{si\text{-}GDF11}$ stained with TMRM under fluorescence microscope to measure mitochondrial membrane potential (ψMt). Scale bar = 100μm. MSCs treated with either FCCP (50 μmol/L) or oligomycin (10 μmol/L) were served as negative and positive controls, respectively. Graphs show relative MFI of si-NC and si-GDF11 divided by the difference of MFI between Oligomycin and FCCP (n=8). Three independent experiments were repeated. Data were shown as mean ± SD. * *P<0.0*5 vs si-NC.
