# Supplementary material for: GDF11 enhances therapeutic efficacy of mesenchymal stem cells for myocardial infarction via YME1L‐mediated OPA1 processing
Source: Stem Cells Transl Med. 2020 Jun 9;9(10):1257–71. doi: 10.1002/sctm.20-0005 (PMC7519765; doi:10.1002/sctm.20-0005)
Supplement: Supplementary file 8 — Figure S8. Supporting information [file SCT3-9-1257-s019.pdf]

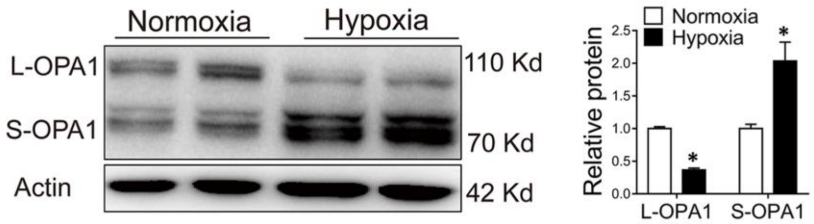

Figure. S8 Hypoxia promoted OPA1 proteolytic cleavage. Representative immunoblots and densitometric quantification for the expression of OPA1 cleavage product (L-OPA1 and S-OPA1) in MSCs under normoxia and hypoxia conditions. Densitometric analysis of L-OPA1 and S-OPA1 in right (n=4). Three independent experiments were repeated. Data were shown as mean ± SD. * *P<0.0*5 vs Normoxia.
