# Supplementary material for: GDF11 enhances therapeutic efficacy of mesenchymal stem cells for myocardial infarction via YME1L‐mediated OPA1 processing
Source: Stem Cells Transl Med. 2020 Jun 9;9(10):1257–71. doi: 10.1002/sctm.20-0005 (PMC7519765; doi:10.1002/sctm.20-0005)
Supplement: Supplementary file 9 — Figure S9. Supporting information [file SCT3-9-1257-s020.pdf]

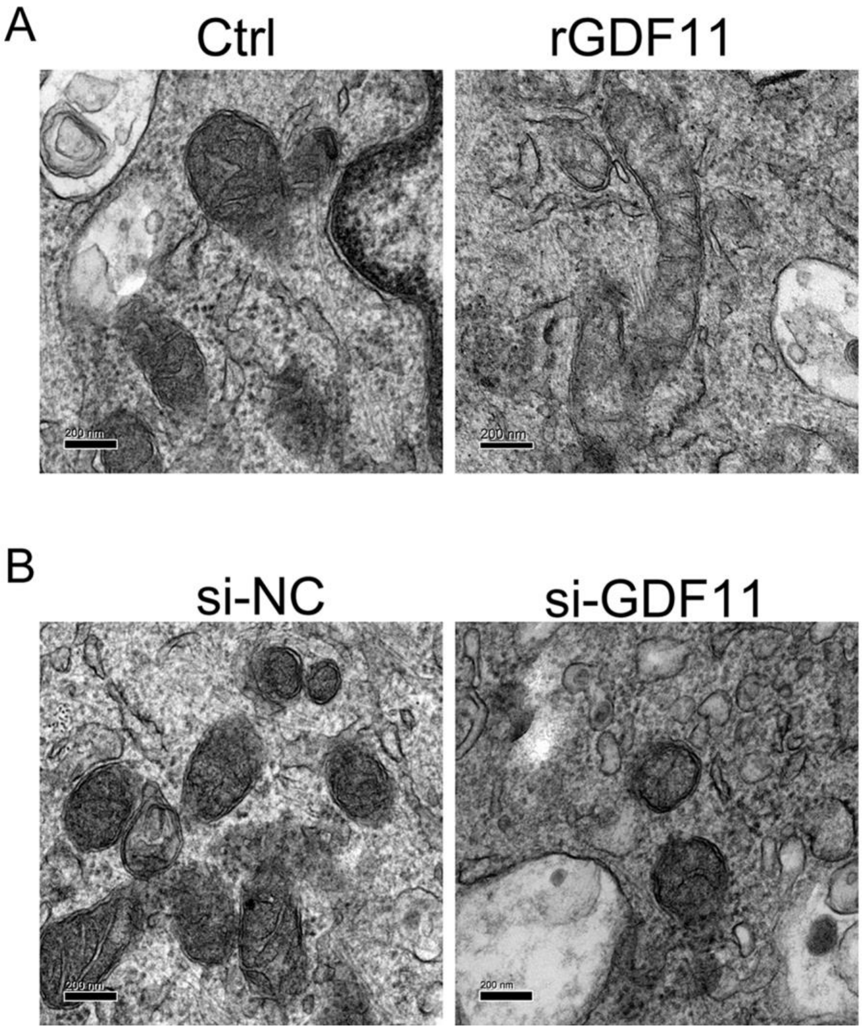

Figure. S9 GDF11 protected mitochondrial cristae under hypoxic condition. **A.** Representative images of the mitochondrial cristae in MSCs pretreated with or without rGDF11 under hypoxia conditions (magnification was set at ×26,500). Scale bars = 200nm. **B.** Representative images of the mitochondrial cristae in MSCs transfected with siRNA-NC or siRNA-GDF11 under hypoxia conditions (magnification was set at ×26,500). Scale bars = 200nm.
