# Supplementary material for: GDF11 enhances therapeutic efficacy of mesenchymal stem cells for myocardial infarction via YME1L‐mediated OPA1 processing
Source: Stem Cells Transl Med. 2020 Jun 9;9(10):1257–71. doi: 10.1002/sctm.20-0005 (PMC7519765; doi:10.1002/sctm.20-0005)
Supplement: Supplementary file 10 — Figure S10. Supporting information [file SCT3-9-1257-s001.pdf]

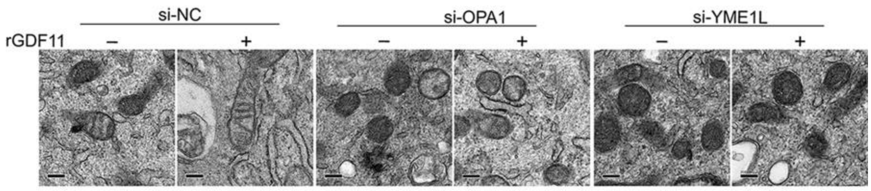

Figure. S10 GDF11 regulated mitochondrial morphologies dependent on the *i*-AAA (ATPases associated with diverse cellular activities) protease YME1L and OPA1. Representative images of the mitochondrial cristae in each group of MSCs transfected with siRNA-OPA1 or siRNA-YME1L for 48h and then incubated with rGDF11 (50ng/ml) for 24h and then exposed to hypoxia conditions for 48h (magnification was set at ×26,500). Three independent experiments were repeated. Scale bars = 200nm.
