# Supplementary material for: GDF11 enhances therapeutic efficacy of mesenchymal stem cells for myocardial infarction via YME1L‐mediated OPA1 processing
Source: Stem Cells Transl Med. 2020 Jun 9;9(10):1257–71. doi: 10.1002/sctm.20-0005 (PMC7519765; doi:10.1002/sctm.20-0005)
Supplement: Supplementary file 11 — Figure S11. Supporting information [file SCT3-9-1257-s002.pdf]

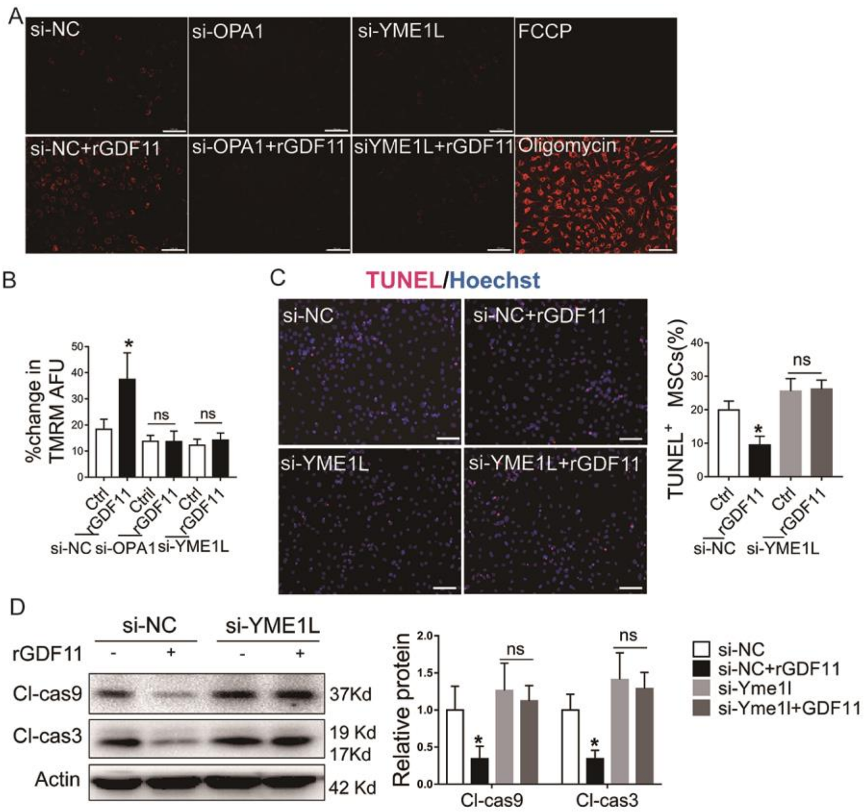

Figure. S11 GDF11 regulated anti-apoptosis dependent on the *i*-AAA protease YME1L. **A, B.** Fluorescence images of MSCs stained with TMRM were used to measure mitochondrial membrane potential. Scale bar =100μm. Quantification of fluorescence intensities was as described in Fig 3F and presented in B (n=8). **C.** Representative images of TUNEL staining (red) of MSCs treated as described in (A) for apoptotic cells. DAPI staining for nuclei. Scale bar =50μm. Quantification of apoptotic cells was presented as ratio of TUNEL-positive nuclei over the total nuclei from 8 to 10 randomly selected fields in each sample. **D.** Cleaved-caspase 3 and cleaved-caspase 9 protein expression levels were assessed by Western blot in MSCs^{siNC}, MSCs$^{siNC+rGDF11}$, MSCs$^{si-Yme1l}$ and MSCs$^{siYme1l+rGDF11}$ (n=3). β-actin served as a control. Data were shown as mean ± SD. * *P<0.0*5 vs si-NC.

1
