# Supplementary material for: GDF11 enhances therapeutic efficacy of mesenchymal stem cells for myocardial infarction via YME1L‐mediated OPA1 processing
Source: Stem Cells Transl Med. 2020 Jun 9;9(10):1257–71. doi: 10.1002/sctm.20-0005 (PMC7519765; doi:10.1002/sctm.20-0005)
Supplement: Supplementary file 12 — Figure S12. Supporting information [file SCT3-9-1257-s003.pdf]

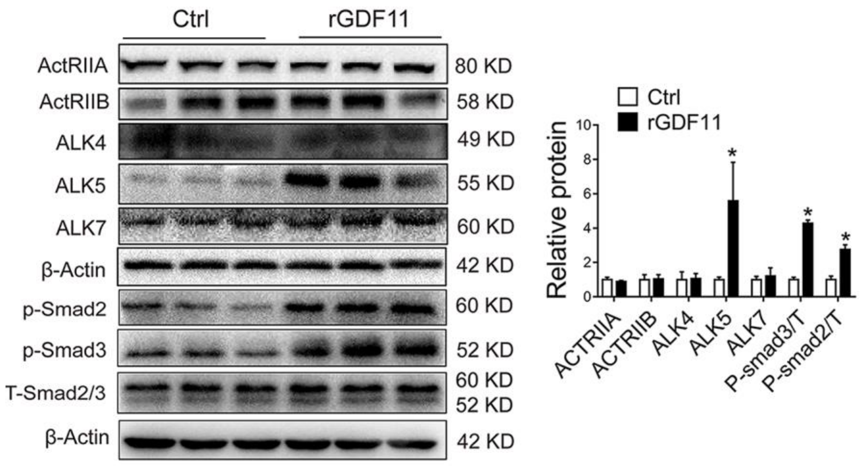

Figure. S12 GDF11 activated Smad2/3 phosphorylation in MSCs. Representative immunoblots and densitometric quantification for the expression of GDF11 receptor (ALK4, ALK5, ALK7, ActRIIA, ActRIIB) and Smad2/3 pathway members (p-Smad2/3 and T-Smad2/3) in MSCs under hypoxic condition (n=3). β-Actin served as control. Data were shown as mean ± SD. * $P<0.05$ vs Ctrl.
