# Supplementary material for: GDF11 enhances therapeutic efficacy of mesenchymal stem cells for myocardial infarction via YME1L‐mediated OPA1 processing
Source: Stem Cells Transl Med. 2020 Jun 9;9(10):1257–71. doi: 10.1002/sctm.20-0005 (PMC7519765; doi:10.1002/sctm.20-0005)
Supplement: Supplementary file 13 — Figure S13. Supporting information [file SCT3-9-1257-s004.pdf]

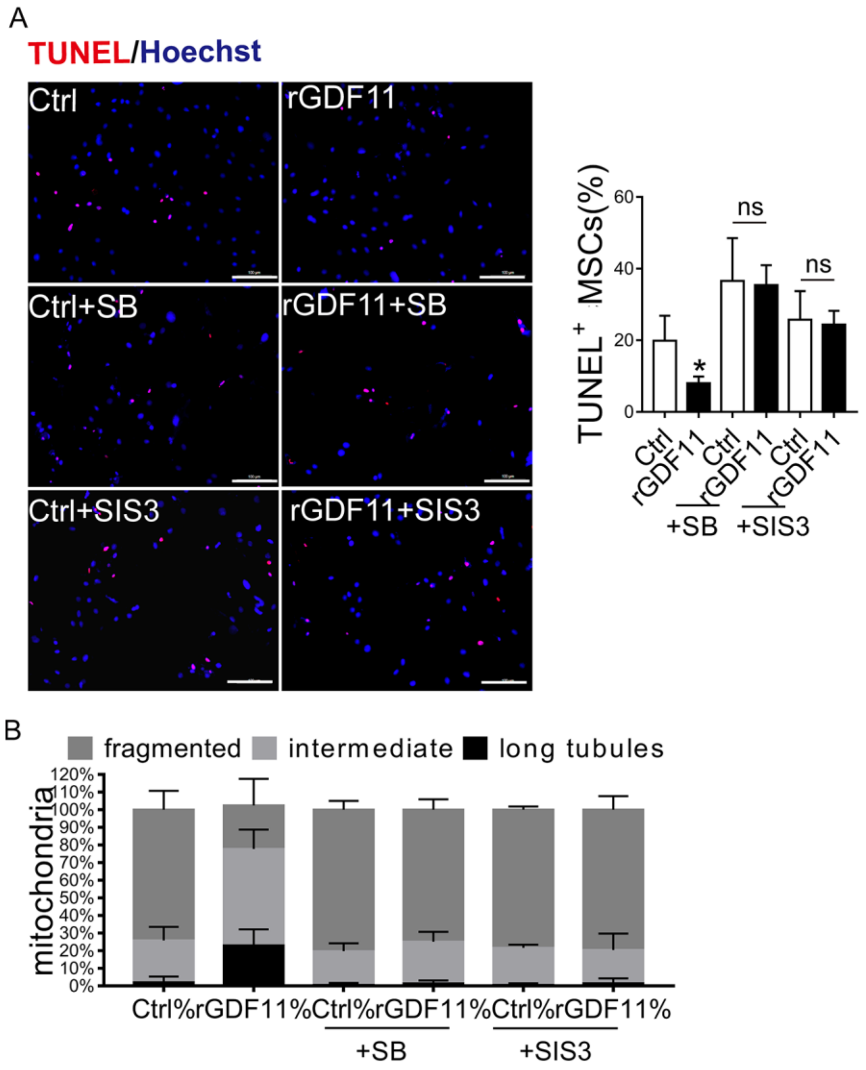

Figure. S13 GDF11 protected MSCs from hypoxia-induced apoptosis through TGF-β-smad2/3 pathway. **A.** TUNEL staining of MSCs were assessed cellular apoptosis. MSCs were treated SB431542 or SIS3 for 30 min and then incubated with rGDF11 (50ng/ml) for 24h then exposed to hypoxia conditions for 48h. Scale bar = 50μm. Quantification of apoptotic cells was presented as ratio of TUNEL-positive nuclei over the total nuclei from 8 to 10 randomly selected fields in each sample. **B.** Quantification of mitochondrial distribution of mitochondria according to their length: long tubules (＞ 0.65μm), intermediate (≤0.65μm, ≥0.32μm) and fragmented (＜0.32μm). Data were

shown as mean ± SD. * $P<0.05$ vs Ctrl.
