# Supplementary material for: GDF11 enhances therapeutic efficacy of mesenchymal stem cells for myocardial infarction via YME1L‐mediated OPA1 processing
Source: Stem Cells Transl Med. 2020 Jun 9;9(10):1257–71. doi: 10.1002/sctm.20-0005 (PMC7519765; doi:10.1002/sctm.20-0005)
Supplement: Supplementary file 14 — Figure S14. Supporting information [file SCT3-9-1257-s005.pdf]

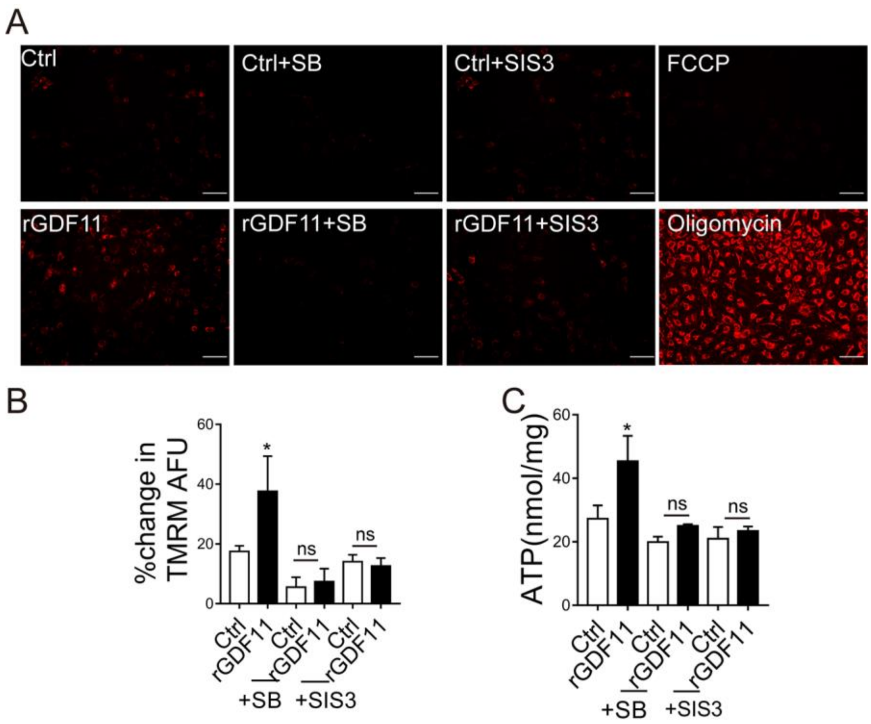

Figure. S14 GDF11 regulated mitochondrial functions dependent on TGF-β-Smad2/3 pathway. **A, B.** Images of MSCs which were treated with TGFβR1 inhibitor SB4431542 or p-Smad3 inhibitor SIS3 for 30 min, and incubated with rGDF11(50ng/ml) for 24h and then exposed to hypoxia condition for 48h. stained with TMRM under fluorescence microscope were used to measure mitochondrial membrane potential as described in the legend of Fig 3 (n=8). Scale bar =100μm. **C.** Intracellular ATP levels were determined in the indicated cells under hypoxic conditions. ATP levels were calibrated with protein content (n=3). Data were shown as mean ± SD. * $P<0.05$ vs Ctrl.
