# Supplementary material for: GDF11 enhances therapeutic efficacy of mesenchymal stem cells for myocardial infarction via YME1L‐mediated OPA1 processing
Source: Stem Cells Transl Med. 2020 Jun 9;9(10):1257–71. doi: 10.1002/sctm.20-0005 (PMC7519765; doi:10.1002/sctm.20-0005)
Supplement: Supplementary file 15 — Figure S15. Supporting information [file SCT3-9-1257-s006.pdf]

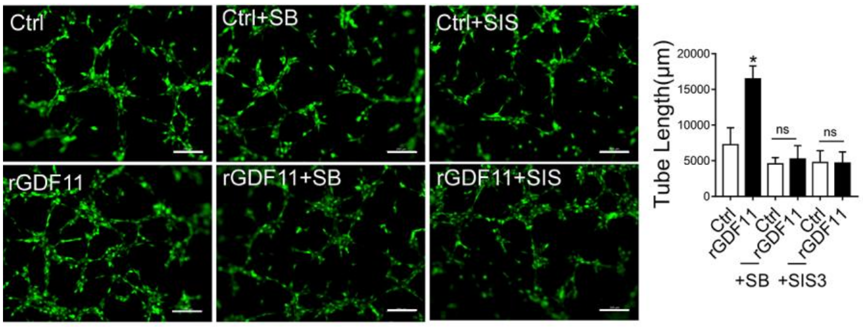

Figure. S15 GDF11 promotes the paracrine effect of MSCs dependent on TGF-β-Smad2/3 pathway. Representative images of the tube formation of HUVECs cultured with conditioned medium from MSCs in different groups. Conditioned mediums were derived from MSCs treated with SB431542 or SIS3 for 30 min and then incubated with rGDF11 (50ng/ml) for 24h or only DMEM and rGDF11(50ng/ml) for 24h. Scale bar =100μm. The quantification of tube formation was shown in bar graphs (n=10). Data were shown as mean ± SD. * *P<0.0*5 vs Ctrl.
