# Supplementary material for: GDF11 enhances therapeutic efficacy of mesenchymal stem cells for myocardial infarction via YME1L‐mediated OPA1 processing
Source: Stem Cells Transl Med. 2020 Jun 9;9(10):1257–71. doi: 10.1002/sctm.20-0005 (PMC7519765; doi:10.1002/sctm.20-0005)
Supplement: Supplementary file 16 — Figure S16. Supporting information [file SCT3-9-1257-s007.pdf]

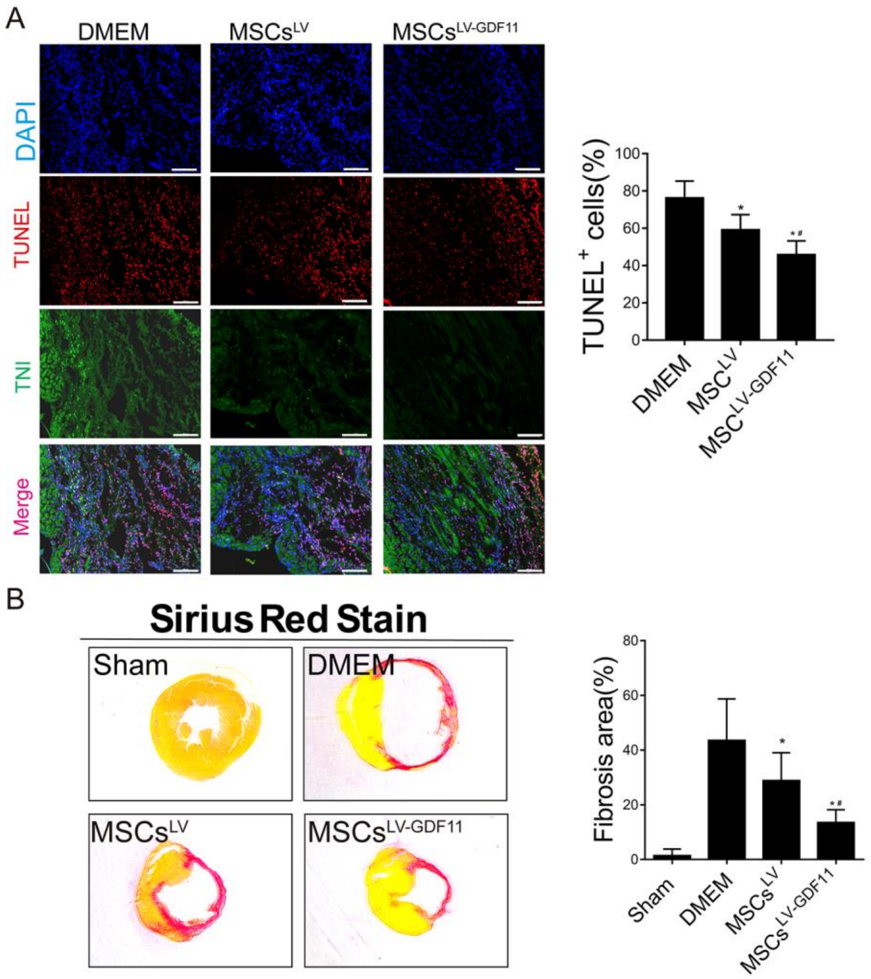

Figure. S16 MSCs pretreated with GDF11 alleviates cardiac apoptosis after MI. **A.** Cardiac cell apoptosis of the peri-infarct zone at 3 day after MI in was determined by TUNEL staining(red), nuclei stained with DAPI (blue) and myocardium stained with troponin I (TNI, green) (n=6 for each group). Scale bar =100μm. Quantification of apoptotic cells by TUNEL-positive nuclei. **B.** After day 28 post MI, the scar size was quantified by Sirius red staining. Quantification of the scar size expressed as the ratio of the length of collagen deposited area over the perimeter of the left ventricle. Five samples were analyzed for each group. Data are shown as mean ±SD. $^{*}P<0.05$ vs. DMEM, $^{\#}P<0.05$ vs. $MSCs^{LV}$.
