# Supplementary material for: GDF11 enhances therapeutic efficacy of mesenchymal stem cells for myocardial infarction via YME1L‐mediated OPA1 processing
Source: Stem Cells Transl Med. 2020 Jun 9;9(10):1257–71. doi: 10.1002/sctm.20-0005 (PMC7519765; doi:10.1002/sctm.20-0005)
Supplement: Supplementary file 17 — Figure S17. Supporting information [file SCT3-9-1257-s008.pdf]

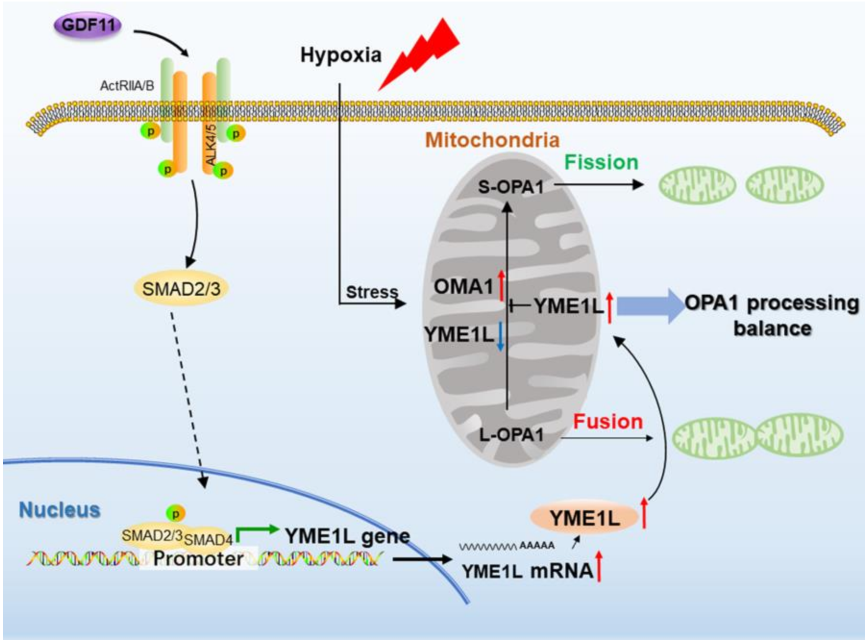

Figure. S17 Possible molecular pathway that GDF11 protects MSCs from hypoxia stress. Mitochondria are dynamic organelles undergoing continuous fluctuation between fission and fusion events. Under hypoxic stress, mitochondrial dynamic is imbalance reflected by more mitochondrial fission events and fusion events reduced, and directly affect the MSCs survival. Optic Atrophy 1 (OPA1) is crucial for inducing mitochondrial inner membrane fusion and maintaining cristae structure. Two mitochondrial inner membrane proteases, OMA1 and YME1L, are essential regulators of OPA1 processing. YME1L accelerates the mitochondrial fusion, whereas OMA1 promotes the mitochondrial fission. Hypoxia leads to more OMA1 activation and inactivation of YME1L, produce redundant S-OPA1 in response to stress, which breaks down the rational ratio between L-OPA1 and S-OPA1. However, GDF11 induces fusion of mitochondrial networks in hypoxic MSCs through ALK4/5-p-Smad2/3 pathway. GDF11 plays an important role in the regulation of mitochondrial dynamics balance

through increasing the expression of YME1L. Increased YME1L rebalances OPA1 processing and inhibits mitochondrial fragmentation in the MSCs under hypoxia stress condition.
