# Supplementary material for: GDF11 enhances therapeutic efficacy of mesenchymal stem cells for myocardial infarction via YME1L‐mediated OPA1 processing
Source: Stem Cells Transl Med. 2020 Jun 9;9(10):1257–71. doi: 10.1002/sctm.20-0005 (PMC7519765; doi:10.1002/sctm.20-0005)
Supplement: Supplementary file 18 — Data S1. Supporting information [file SCT3-9-1257-s009.docx]

**Supplemental Materials and Methods**

**Ethics Statement and animal procedures**

All animal experiments were performed with approval of the Animal Ethics Committee of Zhejiang University, which complies with the Guide for the Care and Use of Laboratory Animals, 8^th^ edition published by the US National Institutes of Health (NIH Publication, 2011). All mice were housed in stainless steel cages with sawdust bedding. They were kept at 23 ± 1 °C, humidity 55 ± 5%, under a 12h dark/light cycle and were allowed unlimited food and water. To harvest tissues like heart for isolation of MSCs, adult mice were anesthetized by isoflurane inhalation and sacrificed by cervical dislocation.

**Cell isolation and culture**

Mouse cardiac MSCs (MSCs) were isolated from hearts of mouse (C57BL/6) at 8 to 12-week by procedures as previously described [1, 2], and were provided by Dr. Yaoliang Tang at Medical College of Georgia, Augusta University (Augusta, GA, USA). Briefly, ventricular heart tissues were minced into 1 mm^3^ size and digested with 0.1% collagenase IV and 1 U/mL Dispase in DMEM/F-12. The digested heart tissue was seeded into a 6-well plate coated with fibronectin/gelatin (0.5 mg fibronectin in 100 mL 0.1% gelatin). The grown out round, phase-bright cells migrated from adherent explants were collected and undergone hematopoietic cell depletion using the mouse hematopoietic lineage depletion cocktail kit (Stem Cell Technologies) by magnetic activated cell sorting (MACS). These cells were cultured in complete medium (DMEM/F12 containing 10% fetal bovine serum (FBS), 200 mmol/L l-Glutamine, 55 nmol/L β-mercaptoethanol and 1% MEM non-essential amino acid) and used as MSCs for whole study.

Human umbilical vein endothelial cells (HUVECs) and H9C2 were provided by All Cells (Shanghai, China). HUVECs were cultured in Dulbecco’s modified Eagle’s medium (DMEM) with low glucose, supplemented with 10% FBS and 10 U/mL streptomycin-penicillin. H9C2 myoblasts were maintained in DMEM with high glucose, supplemented with 10% FBS. Cell lines were authenticated routinely and were mycoplasma free. MSCs were plated in a 6-well plate at a density of 1×10^6^ cells with complete growth medium. The culture media were replaced with 2 ml serum-free growth media before hypoxia treatment. Hypoxia (0.5% O_2_, 5% CO_2,_ 37^o^C) treatment was achieved with a ProOx-C-chamber system (Biospherix, Redfield, NY) for 48h. Normoxic-treated MSCs (21% O_2_, 5% CO_2_, 37℃) were used as a control.

**Flow cytometry analysis for cell characterization**

MSCs at passages 3-8 were used and identified by flow cytometric analysis as described previously[3]. (PE)-conjugated antibodies against the specific surface moleculars: CD44(#553135), CD105(#562762), CD29(#555479), FLK-1(#561052), CD31(#553373), CD45(#561087) (all from BD Biosciences, San Jose, CA, USA)) were used to culture with cells for 1 hour at room temperature, and the expression of cell surface markers was analyzed using ﬂow cytometry with a BD FACS Count II Flow Cytometer (BD Biosciences, San Jose, CA, USA) (Fig. S1).

**Lentiviral vector construction and cell transduction**

Lentiviral vector expressing mouse GDF11 tagged with ﬂag (Flag-GDF11) was provided by Vigene Bioscience Co., Ltd. (Jinan, China). Lentiviral vector containing no GDF11 gene served as controls. MSCs were transduced with lentiviral vectors as reported previously[4]. In brief, MSCs were cultured on a 6-well plate with an initial 5×10^4^ cells per well in 2 ml DMEM/F12 medium with 10% FBS. After 24h culture, medium was replaced with fresh DMEM/F12 with 10% FBS, and 20 μl viral vector was added along with polybrene (final concentration of 8ug/ml, Sigma) to each multiplicitie of infection (MOI) of 50. After 8h incubation, the supernatant was replaced with fresh DMEM/F12 medium with 10% FBS. Cells were cultured for another 48h.

**Cell siRNA transfection**

Silence interference RNA (siRNA) targeting mouse GDF11 (si-GDF11), OPA1 (si-OPA1), YME1L (si-YME1L) and control siRNA (si-NC) were synthesized by Guangzhou RiboBio Co., Ltd. (Guangzhou, China). siRNA transfection was conducted using Lipofectamine 3000 (Invitrogen, CA, USA). After 24h incubation, the transfected medium was changed with complete medium and then cultured for another 48h. After OPA1 and YME1L were silenced by siRNA, MSCs were further treated with rGDF11 or control for an additional 24h, followed by exposure to hypoxic condition for 48h. The gene primers are shown in Table S1.

**Cell proliferation**

Cell proliferation was examined using 5-ethynyl-2′-deoxyuridine (EdU, RiboBio, Guangzhou, China) staining according to the manufacturer’s introduction. MSCs were plated in 48-well plates at a density of 2×10^4^ cells/well, then treated with recombinant GDF11 (rGDF11, 50ng/ml) (Peprotech, Rocky Hill, USA) in DMEM/F12 medium with 2% FBS for 24h. Cells were washed with PBS, then incubated in serum-free DMEM containing 10μmol/L EdU for 5h. Cells were fixed, then underwent Apollo staining and DNA staining with hochest33342 (Beyotime Biotechnology, Shanghai, China) according to the manufacturer’s instructions to detect the number of cycling cells during the EdU treatment. The cells were imaged using fluorescence microscopy, and the number of proliferating cells was averaged to calculate the labeling index.

**Cell survival**

Cell survival was examined using Cell Count Kit-8 (CCK-8) (Bestbio, Shanghai, China) according to the manufacturer’s introduction. MSCs were cultured on a 96-well plate with an initial 2,000 cells in 100 μl DMEM/F12 medium with 10% FBS per well. MSCs were treated with rGDF11 (50ng/ml) for 24h in 100 μl DMEM/F12 medium with 2% FBS. Then medium was replaced with fresh DMEM/F12, MSCs were exposed to hypoxic condition for 48h. The mixture of 100 μl medium with 10μl CCK8 solution was added into each well. After incubation for 3h at 37 ℃, the absorbance at 450 nm was measured by microplate reader (Bio-Rad, Berkeley, CA, USA).

**Preparation of MSCs-Conditioned Medium and Measurement of Vascular Endothelial Growth Factor A (VEGFA)**

Conditioned medium was generated as previously described[5]. MSCs were pretreated with rGDF11(50ng/ml), TGFβ type Ⅰ receptor inhibitor SB431542(1nm) and Smad3 inhibitor SIS3 (3nm) for 24h or overexpressed GDF11 by transduced with lentivirus vector for 48h or silenced GDF11 by transfected with siRNA for 48h. Then the medium was replaced with fresh DMEM/F12 without serum and incubated for another 48h. The supernatant was collected and then concentrated 5-fold by Centricon concentrators (Millipore, Billerica, MA, USA) and normalized by an equivalent number of MSCs (1×10^6^ cells). VEGFA levels were determined using a commercial ELISA kit (Cloud-Clone Corp., Wuhan, China) according to the manufacturer’s introduction.

**Matrigel tube formation assay**

Matrigel assay was used to analyze the ability of MSCs-conditioned media to promote tube formation of HUVECs in vitro. DMEM/F12 with no serum was used as a control. Matrigel (50 μl/well, BD Biosciences, CA, USA) was put into 96-well plate at 4°C, then incubated for 30min at 37°C. HUVECs suspended at 2.0×10^4^ cells/well in 100 μl conditioned medium from above mentioned were seeded on the Matrigel-coated 96-well and examined for tube formation by phase-contrast microscopy (Olympus) or fluorescence microscopy (Leica, Wetzlar, Germany) 3-5 h later. The conditioned medium had been normalized by an equivalent number of MSCs (1×10^6^ cells). The length of the tube structure was quantiﬁed by ImageJ software.

**TUNEL staining**

Tissue or cell samples were ﬁxed with 4% paraformaldehyde and permeabilized with 0.2-0.5% Triton for 10 min each, and then, incubated with TUNEL (Beyotime Biotechnology, Shanghai, China) reaction compound for 60 min at 37°C in the dark according to the manufacturer’s instructions. Nuclei were stained with hochest33342. The apoptotic ratio was calculated as TUNEL-positive cells in total cells/nuclei. The images were obtained from 6 to 10 randomly selected ﬁelds in each sample or per well.

**Transmission electron microscopy (TEM)**

TEM was applied to detect the mitochondrial network ultrastructure of

MSCs. In brief, the samples were ﬁxed with 2.5% glutaraldehyde for >4h. The specimens were post-fixed with 1% OsO_4_ for 1-2h after washing three times with phosphate-buffered saline. Then, the specimens were dehydrated by an ethanol gradient, followed by acetone for overnight infiltration. Furthermore, the specimens were embedded in Spurr resin and sectioned in Leica EM UC7 (Leica, Wetzlar, Germany). The sections were stained with uranyl acetate and alkaline lead citrate, and the image procured by Hitachi Model H-7650 TEM. Images were obtained randomly to measure the mitochondrial ultra-microstructure using the open-source image analysis program ImageJ (NIH) under magnification of ×10,000 and ×26,500.

**Oxygen consumption rate**

Oxygen consumption rate (OCR), an indicator of mitochondrial respiration, was measured in the intact cells as described previously[6]. Three readings were taken after each addition of mitochondrial inhibitor before injection of the subsequent inhibitors, and each parameter were made over a 15 min period. The mitochondrial inhibitors used were ATP synthase inhibitor oligomycin (final concentration: 1 μg/ml), the proton ionophore carbonylcyanide p-trifluoromethoxyphenylhydrazone (FCCP; final concentration 1 μM), and antimycin A, a complex III inhibitor, and rotenone, a complex I inhibitor (final concentration, 1 μM). Mitochondrial function parameters were determined using mitochondrial inhibitors as modulators to determine the number of bioenergetic and mitochondrial function parameters, including basal respiration, ATP turnover rate, proton leak, and maximal and spare respiratory capacity. OCR was measured by using OROBOROS Oxygraph-2k at 30°C (Oroboros Instruments, Austria).

**ATP measurement**

Total cellular ATP content of MSCs under hypoxic stress was determined by using a luminescence ATP detection kit in accordance with the manufacturer’s instructions (Beyotime Biotechnology, Shanghai, China). The total cellular ATP content was determined by running an internal standard and expressed as nmol/mg.

**Mitochondrial membrane potential**

Mitochondrial membrane potential was measured after MSCs were stained with tetramethyl rhodamine methyl ester (TMRM, 200 nmol/L) for 30 min at 37 ℃ in a humidified incubator with 5% CO_2_. The cells were also incubated with oligomycin (10μM) and FCCP (50μM) for 30 min as a positive and negative controls, respectively. Cells were washed with PBS three times and covered with 200μl PBS. Images were acquired under fluorescence microscope (400×) at 549 nm for excitation and 573 nm for emission. TMRM fluorescent intensity was counted in TMRM channel. Relative mean of fluorescence intensity (MFI) of Control or rGDF11 divided by the difference of MFI between Oligomycin and FCCP was obtained using ImageJ software.

**Isolation of cytosolic and mitochondrial fractions**

Isolation of cytosolic and mitochondrial fraction was conducted by using Mitocondria Isolation Kit for Cultured Cells (Beyotime Biotechnology, Shanghai, China) following the manufacturer’s protocol. In brief, 1×10^7^ cells with indicated treatments were collected after centrifugation of the harvested cell suspension, and then mitochondria isolation regent with Phenylmethylsulfonyl Fluoride was added into the cell pellets. The cell resuspension was homogenized by glass homogenizer 33 times, then centrifuged at 600×g for 10 min at 4 ℃. The supernatant was transferred to a new tube and centrifuged at 11,000 ×g for 10 min at 4 ℃. The supernatant was transferred to a new tube, and the pellet contained the isolated mitochondria. Finally, cytosolic fraction was derived from the supernatant centrifuged at 12,000 ×g for 10 min at 4 ℃. Moreover, the purity of the mitochondria was ensured by detecting the expression of tubulin that was the loading control of cytosolic protein.

**Chromatin immunoprecipitation (ChIP) assay**

Promoter of YME1L was analyzed using Jaspar software (<http://jaspar.genereg.net/>) to identify putative binding sequences by Smad2/3. Chromatin immunoprecipitation (ChIP) was performed using Simple ChIP^®^ Plus Sonication Chromatin IP Kit (Cell Signaling Technology, USA) according to the manufacturer’s instruction with Smad2/3 antibody (Cell Signaling Technology, USA). Rabbit IgG (Cell Signaling Technology, USA) was used as negative control and input DNA without IP as positive control. MSCs were treated with 0.1% BSA (Control) or 50 nmol/ml rGDF11 for 24h. Approximately 1×10^7^ cells were evaluated for each sample. The purified DNA and input genomic DNA were analyzed by real time PCR. Reaction mixtures were cycled with an initial melt step at 95°C for 30 seconds and then 30 cycles of 95°C for 15 seconds, 60°C for 30 seconds, and 72°C for 10 seconds, followed by 95°C for 15 seconds, 60°C for 1min and 95°C for 15 seconds. Products were analyzed by electrophoresis on a 1.5% gel. Primer sequences of YME1L promoter were shown in online resource Table S1.

**Murine myocardial infarction and cell delivery**

Myocardial infarction model was performed on male mice (C57BL/6, 8-10 week-old, 20–25 g weight, Shanghai SLAC Laboratory Animal Co.,Ltd, Shanghai, China). Mice were anesthetized by intraperitoneal injection of 100mg/kg ketamine combined with 10mg/kg xylazine and ventilated via tracheal intubations connected to a rodent ventilator. MI surgery was conducted by ligation of left anterior descending coronary artery as described previously[7]. MSCs^LV^ or MSCs^LV-GDF11^ (both 5×10^5^ cells, in 20 μl DMEM) or DMEM alone were injected at 5 sites around the border zone of infarcted heart immediately after left anterior descending ligation using a 31-gauge Hamilton syringe. The Sham group underwent the same surgical procedures except for the permanent ligation step. To ensure adequate power to detect a pre-specified effect, the sample size was chosen using the Power and Sample Size Program (<http://biostat.mc.vanderbilt.edu/PowerSampleSize>).

**Ultrasound Analysis of Cardiac Function**

Echocardiography was performed to evaluate cardiac function before (baseline, day -1) and after MI (day 3, 7, 14, and 28) as reported previously[4]. Transthoracic two-dimensional M-mode were used to measure left ventricular ejection fraction (LVEF), and left ventricular fractional shortening (LVFS) with the Vevo 2100 system (VisualSonics, Toronto, Canada). This procedure was repeated 3 times with use of the same equipment by the same examiner. All procedures and analysis were performed by a researcher who was blinded to treatment.

**Histochemical Staining**

Heart tissues were harvested at day 3 and 28 post MI for histological analysis as described previously[5]. Heart tissues were either embedded in Tissue Tek O.C.T. compound (Sakura Finetek USA Inc., CA) or in parafﬁn after fixed in neutral buffered formalin. The scar size was evaluated by Sirius Red staining at day 28 post MI. Scar areas were calculated by the sum of the endocardial and epicardial length of the infarct zone in proportion to the total length of the endocardial and epicardial left ventricle using Image-Pro-Plus software (Media Cybernetics, Rockville, MD, USA). Immunofluorescence staining was taken to detect retained MSCs at day 3 post MI, capillaries and small arteries at day 28 post MI using specific antibodies against GFP, CD31, and α-SMA, respectively. In brief, frozen tissues slices were fixed with 4% paraformaldehyde (PFA), permeabilized with 0.2% TritonX-100 for 10 min, blocked by 5% bovine serum albumin (BSA) and incubated with primary antibodies overnight at 4°C, followed by incubation with secondary antibody for 1h at room temperature. Nuclei were counterstained with DAPI. The samples were analyzed using a fluorescence microscope (Leica, Wetzlar, Germany). Positively stained cells were counted in three sections per heart, five high-power fields (HPFs) per section. The antibodies (Ab) are listed: GFP primary Ab (1:200, Abcam, ab13970, Cambridge, MA, USA); α-SMA (1:200, Abcam, ab32575, Cambridge, MA, USA); CD31 (1:200, BD Bioscience, #553370, San Jose, CA, USA); Cardiac Troponin I (1:200, Abcam, #ab47003); anti-rabbit, anti-goat and anti-mouse secondary antibody (1:200, Abcam).

**Quantitative real-time PCR**

The RNA extraction and quantitative real-time PCR procedure were carried out as previously reported[5]. Total RNA was isolated by Trizol reagent (TaKaRa, Dalian, China) according to the manufacturer’s protocol. Gene expression levels were determined by qRT-PCR using SYBR Green (TaKaRa, Dalian, China) and normalized to those of 18s, which was used as an internal control. Data were calculated by the comparative 2^-ΔΔCt^ methods, and the gene primers are shown in Table S1.

**Western Blot**

Western blot was performed as described previously[8]. Cultured or treated cells were lysed by the RIPA lysis buffer (Beyotime, Jiangsu, China) on ice. Protein concentrations were quantified by BCA protein assay (Bio-Rad, Berkery, CA, USA). The immunoblots were probed with appropriate primary overnight at 4°C followed by incubation with the corresponding secondary antibodies at room temperature for 1 h. The blots were infiltrated with ECL (Bio-Rad, USA) and detected by ChemiDoc™ MP Imaging System (Bio-Rad, USA). The densitometry of target bands was normalized to internal control β-actin. The antibodies are listed as follow up: GDF11(1:1000, R&D systems, #AF1958, MTC, USA); P-smad2 (ser465/467) (1:1000, Cell Signaling Technology, #18338T, Danvers, MA, USA); P-smad3 (ser423/425) (1:1000, Cell Signaling Technology, #9529T); Samd3 (1:1000, Cell Signaling Technology, #9523T); Samd2/3 (1:1000, Cell Signaling Technology, #8685T); ActRIIA (1:1000, Abcam, #ab135634); ActRIIB (1:1000, Abcam, #ab76940); ALK4 (1:1000, Proteintech, #10086-1-AP, Wuhan, China); ALK5 (1:1000, R&D systems, #AF3025, MTC, USA); ALK7 (1:1000, Proteintech, #12610-1-AP); PGC-1α (1:1000, Abcam, ab54481); Mitofusion-1 (1:1000, Cell Signaling Technology, #14739S); Mitofusion-2 (1:1000, Cell Signaling Technology, #11925S); Drp1 (1:1000, Cell Signaling Technology, #8570S); TOM20 (1:1000, Cell Signaling Technology, #42406S); mono-OPA1 (1:1000, Abcam, ab119485); poly-OPA1 (1:1000, Abcam, ab42364); OMA1 (1:1000, Abcam, ab154949); YME1L (1:1000, Proteintech, #11510-1-AP); cleaved-caspase3 (1:1000, Cell Signaling Technology, #9664S); cleaved-caspase9 (1:1000, Cell Signaling Technology, #9509S); Bcl-2 (1:1000, Cell Signaling Technology, #3498S); Bax (1:1000, Cell Signaling Technology, #5023S); β-actin (1:3000, #R1102-1, Huabio, Hangzhou, China); tubulin (1:3000, Huabio, #M1305-2); anti-rabbit, anti-rat and anti-mouse secondary antibody (1:3000, Huabio, #HA1011, #HA1022, #HA1006).

**References**

1. Tang YL, et al. A novel two-step procedure to expand cardiac Sca-1+ cells clonally. Biochem Biophys Res Commun (2007); 359**,** 877-883.

2. Tang YL, et al. Hypoxic preconditioning enhances the benefit of cardiac progenitor cell therapy for treatment of myocardial infarction by inducing CXCR4 expression. Circ Res (2009); 104**,** 1209-1216.

3. Chen L, et al. Two-step protocol for isolation and culture of cardiospheres. Methods Mol Biol (2013); 1036**,** 75-80.

4. Yang F, et al. Leptin increases mitochondrial OPA1 via GSK3-mediated OMA1 ubiquitination to enhance therapeutic effects of mesenchymal stem cell transplantation. Cell Death Dis (2018); 9**,** 556.

5. Wang K, et al. Enhanced Cardioprotection by Human Endometrium Mesenchymal Stem Cells Driven by Exosomal MicroRNA-21. Stem Cells Transl Med (2017); 6**,** 209-222.

6. Nan J, et al. TNFR2 Stimulation Promotes Mitochondrial Fusion via Stat3- and NF-kB-Dependent Activation of OPA1 Expression. Circ Res (2017); 121**,** 392-410.

7. Zhu J, et al. Myocardial reparative functions of exosomes from mesenchymal stem cells are enhanced by hypoxia treatment of the cells via transferring microRNA-210 in an nSMase2-dependent way. Artif Cells Nanomed Biotechnol (2018); 46**,** 1659-1670.

8. Ma Q, et al. Profound Actions of an Agonist of Growth Hormone-Releasing Hormone on Angiogenic Therapy by Mesenchymal Stem Cells. Arterioscler Thromb Vasc Biol (2016); 36**,** 663-672.
