# Supplementary material for: GDF11 enhances therapeutic efficacy of mesenchymal stem cells for myocardial infarction via YME1L‐mediated OPA1 processing
Source: Stem Cells Transl Med. 2020 Jun 9;9(10):1257–71. doi: 10.1002/sctm.20-0005 (PMC7519765; doi:10.1002/sctm.20-0005)
Supplement: Supplementary file 19 — Table S1. Sequences of primers and siRNAs used in this study [file SCT3-9-1257-s010.docx]

**Table. S1** Sequences of primers and siRNAs used in this study.

| **Gene** | **Primer sequence 5`-3`** |
| --- | --- |
|  | **Forward Reverse** |
| *GDF11*  CTACCACCGAGACGGTCATAA CCGAAGGTACACCCACAGTT  *Drp1* GCCTCAGATCGTCGTAGTGG TGCTTCAACTCCATTTTCTTCTCC  *Fis* CAGTGTTGCGTGTTAAGGGATG TTCAAAATTCCTTGCAGCTTCGT  *PGC1α* GTCATGTGACTGGGGACTGT CCAGAGCAGCACACTCTATG  *TFAM* TAGGCACCGTATTGCGTGAG GACAAGACTGATAGACGAGGGG  *ATG5* ACCCCTGAAATGAGTTTTCCAGA AAAGTGAGCCTCAACCGCAT  *Mfn1* CAGGGACGGAGTGAGTGTC TTCTGCCATTATGCACCTGG  *Mfn2* ACCAGCTAGAAACTTCTCCTCT CTTGACGGTGACGATGGAGT  *OPA1* TGACAAACTTAAGGAGGCTGTG CATTGTGCTGAATAACCCTCAA  *OMA1* CAGTGCGCGCTCACGATAA CTCTCTCAGGCAGCAGATCG  *YME1L* GGCAGATGTCGTCGTAGCA TGCTCGACAGGGAGAACATC  *18s* CCCAGTAAGTGCGGGTCATAA CCGAGGGCCTCACTAAACC  *si-NC*  5’UUCUCCGAACGUGUCACGU dTdT3’  *si-GDF11* 5’GAGTCCTAGAGAACACGAA dTdT3’  *si-YME1L* 5’CAAGAUAAAGUUUGAUAAA dTdT3’  *si-OPA1*  5’GCUUACAUGCAGAAUCCUA dTdT3’  *YME1L* AGCGTTAAGTGCGTTTGACC CCCCGGAAGACCATAGGTACA  *Chip,2550*  *YME1L* GTAGCTGTCTTCAGACACTCCAGA AGTTTTAGGGGCTGGTGAGATG  *Chip,473* | |
