# Supplementary material for: GDF11 enhances therapeutic efficacy of mesenchymal stem cells for myocardial infarction via YME1L‐mediated OPA1 processing
Source: Stem Cells Transl Med. 2020 Jun 9;9(10):1257–71. doi: 10.1002/sctm.20-0005 (PMC7519765; doi:10.1002/sctm.20-0005)
Supplement: Supplementary file 20 — Table S2. YME1L promoter sequences that were predicted to be bound by SMAD2/3. [file SCT3-9-1257-s012.docx]

**Table. S2. YME1L promoter sequences that were predicted to be bound by SMAD2/3.**

Model ID Model name Start End predicted site sequence

MA0513.1 SMAD2::SMAD3::SMAD4 473 485 GTGGCTCACAACC

MA0513.1 SMAD2::SMAD3::SMAD4 2550 2562 GACTCTGTCACTT

The putative Smad2/3 binding sequences on YME1L were identified by using Jaspar software.
